# Engineered Extracellular Vesicles Loaded with MiR-100-5p Antagonist Selectively Target the Lesioned Region to Promote Recovery from Brain Damage

**DOI:** 10.1007/s12264-025-01376-6

**Published:** 2025-04-01

**Authors:** Yahong Cheng, Chengcheng Gai, Yijing Zhao, Tingting Li, Yan Song, Qian Luo, Danqing Xin, Zige Jiang, Wenqiang Chen, Dexiang Liu, Zhen Wang

**Affiliations:** 1 Department of Physiology, School of Basic Medical Sciences, Cheeloo College of Medicine, Shandong University, Jinan, 250012 China; 2 Department of Medical Psychology and Ethics, School of Basic Medicine Sciences, Cheeloo College of Medicine, Shandong University, Jinan, 250012 China; 3 Qilu Hospital, Cheeloo College of Medicine, Shandong University, Jinan, 250012 China

**Keywords:** MicroRNAs, Extracellular vesicles, Protein phosphatase 3 catalytic subunit alpha, Neuronal survival, Neonatal hypoxic-ischemic brain damage

## Abstract

Hypoxic-ischemic (HI) brain damage poses a high risk of death or lifelong disability, yet effective treatments remain elusive. Here, we demonstrated that miR-100-5p levels in the lesioned cortex increased after HI insult in neonatal mice. Knockdown of miR-100-5p expression in the brain attenuated brain injury and promoted functional recovery, through inhibiting the cleaved-caspase-3 level, microglia activation, and the release of proinflammation cytokines following HI injury. Engineered extracellular vesicles (EVs) containing neuron-targeting rabies virus glycoprotein (RVG) and miR-100-5p antagonists (RVG-EVs-Antagomir) selectively targeted brain lesions and reduced miR-100-5p levels after intranasal delivery. Both pre- and post-HI administration showed therapeutic benefits. Mechanistically, we identified protein phosphatase 3 catalytic subunit alpha (Ppp3ca) as a novel candidate target gene of miR-100-5p, inhibiting c-Fos expression and neuronal apoptosis following HI insult. In conclusion, our non-invasive method using engineered EVs to deliver miR-100-5p antagomirs to the brain significantly improves functional recovery after HI injury by targeting Ppp3ca to suppress neuronal apoptosis.

**Supplementary information:**

The online version contains supplementary material available at 10.1007/s12264-025-01376-6.

## Introduction

Hypoxic-ischemic brain damage resulting from perinatal asphyxia and hypoxic-ischemic (HI) during the perinatal period is a common form of brain damage and can lead to various neurological disorders [1]. In general, 1 to 3 per 1000 live births suffer from HI brain injury in developed countries, while the rate is higher in developing countries, where ~26 per 1000 live births are diagnosed with this serious disease [2, 3]. Although important progress in neonatal clinical treatment (with the advent of therapeutic hypothermia) has been made over the last decades, HI is still an important contributor to neonatal mortality and to major neurodevelopmental disabilities, including mental retardation, cerebral palsy, learning disabilities, and seizures [3, 4]. Currently, the treatment strategies are restricted due to the lack of knowledge regarding the complicated pathogenesis of HI brain injury. Thus, a better understanding of the mechanisms of neuronal cell death and perinatal brain injury and seeking therapeutic strategies to ameliorate the severity of HI-related brain injury remain important.

MicroRNAs (miRNAs) are a growing class of endogenous non-coding small RNAs with regulatory functions (mainly control of post-transcriptional gene expression) [5]. Data have revealed that miRNAs are abundantly expressed in the central nervous system (CNS), and are involved in a variety of neurological disorders, including HI brain damage [6]. Studies have shown, the miR-100-5p released from apoptotic cortical neurons activates endogenous Toll-like receptors 7/8 (TLR7/8) and serves as their ligand to trigger further neuronal apoptosis that contributes to neurodegeneration [7]. However, the expression, role, and specific regulatory mechanisms of miR-100-5p in HI brain damage have scarcely been discussed. In this study, although administering miR-100-5p antagomir *via* stereotaxic technique is effective for experimental purposes, its clinical translation is hampered due to safety concerns associated with this method.

Addressing these concerns, several researchers have investigated the potential of using extracellular vesicles (EVs) as a targeted delivery system to the brain, aiming to facilitate brain injury repair following HI events [8, 9]. It is well recognized that the blood brain barrier (BBB) is the major obstacle to the delivery of drugs to the CNS. It has been estimated that 98% of small molecules do not cross the BBB [10]. Recently, accumulating evidence has showed that EVs, endogenous lipid membrane vesicles 30–150 nm in diameter that can transport therapeutic drugs (including proteins, lipids, and nucleic acids) and possess biocompatibility, metabolic stability, BBB penetrability, and target specificity, are potential avenues for treating neurological diseases [11–14]. Excitingly, a number of groundbreaking studies have demonstrated that therapeutic nucleic acids (miRNAs, siRNA) can be loaded into modified EVs by electroporation [15]. In order to achieve neuron-specific targeting, rabies virus glycoprotein (RVG) has been engineered to the EV surface *via* fused protein lysosome-associated membrane glycoprotein 2b (Lamp2b)-RVG, which has the potential for achieving brain-specific targeting [16].

We devised a method to deliver small-molecule antagonists of candidate miRNAs to the brain *in vivo* by engineering EVs that harbor these antagonists, thereby unlocking the potential of targeted therapeutic delivery. In our current investigation, we initially delved into the concept of utilizing small molecule antagonist delivery strategies in the context of our ongoing research exploring the functional roles of microRNAs in HI brain injury. We confirmed that the microRNA miR-100-5p is increased in the ipsilateral cortical tissue of mice with HI insult. And we successfully obtained engineered-EVs fused with Lamp2b-RVG (RVG-EVs) for targeted delivery to the brain, and miR-100-5p Antagomir was loaded into RVG-EVs by electroporation. Thus, we were able to experimentally determine the associated functional benefits of miR-100-5p Antagomir in HI-mice.

## Materials and Methods

The entire set of data, methods, and research materials are accessible within the article and its accompanying Data Supplement.

### Animals

The C57BL/6J mice were purchased from Weitong Lihua Experimental Animal Technology Co. Ltd. (Beijing, China). All mice were housed under a controlled photoperiod (12 h light/12 h darkness) with rodent food and water *ad libitum*. All experimental protocols involving animals were approved by the Institutional Animal Care and Use Committee of Shandong University (approval No. ECSBMSSDU2018-2-059) (Shandong, China). Newborn mice were obtained from time-mated pregnant C57BL/6 mice. Each animal was randomly assigned to a control group and those under different experimental conditions using simple randomization. All efforts were made to minimize animal suffering and the number of animals used.

### Hypoxic-ischemic Model of Neonatal Mice

The mouse model of neonatal HI brain damage was created using a modification of the Rice-Vannucci model as previously described [17]. In brief, 7-day-old pups (postnatal day 7, PND7) underwent unilateral right common carotid ligation under isoflurane anesthesia. The incision was closed with sutures, and pups were permitted to recover for 30 min with their dams in their home cages. After that, the neonatal mice were placed in a 37°C constant temperature anoxic chamber with nitrogen-oxygen mixed gas (9.7% O_2_/90.3% N_2_, 5% CO_2_) for 1 h. For the sham group, the blood vessel was separated but no ligation or hypoxia treatments were carried out. All PND7 mice were returned to their mothers and left for the same time until they were euthanized.

### Behavioral Analysis

Short-term neurobehavioral tests were applied 24 h after HI injury by observers who were blind to the experiments [18]. Neonatal mice were subjected to the following tests. (1) Righting reflex (1 and 3 days post-HI): each mouse was placed in the supine position, and the time taken to turn over to the prone position with all four paws in contact with the surface was recorded. The maximum time allowed for each rat was 30 s; (2) Geotaxis reflex (1 and 3 days post-HI): newborn mice were placed head down in the middle of a rough, 30-cm long inclined board (45°). The average time for each group of mice to turn 90° was recorded. The maximum time allowed for each mouse was 60 s; and (3) Cliff avoidance reflex (1, 3, and 7 days post-HI): neonatal mice were positioned with their forepaws on the edge of the platform. Each animal was gently placed on the protruding end of the board with its head down and forepaws off the board. The latency to place both forepaws back on the board was measured with a maximum time allowance of 60 s; (4) Gait reflex (3, 5, and 7 days post-HI): this reflex was defined as the time in seconds required for one forelimb to extend beyond a circle 13 cm in diameter. Pups were observed for up to 30 s [19]. Each mouse was examined three times and the mean value was calculated from the triplicate test results.

Long-term neurobehavioral tests were applied during 28–35 days post-HI. (1) Open field test (OFT): locomotor activity was measured using an HVS2100 automated tracking system (HVS Image, Hampton, UK). Mice were placed in an open field chamber (40 × 40 cm box, no ceiling, and white floor) and allowed to explore for 5 min. The number of crossings and the total distance moved were recorded. (2) Y maze: the Y-maze had three arms (20 cm long × 10 cm wide × 20 cm high) at 120° angles: the start arm (always open), the novel arm, which is blocked at the first trial, but opened at the second trial, and the other arm (always open). In the first trial, one of the arms was blocked and the animal was allowed to explore the other two arms for 5 min. The second trial was conducted 0.5 h later, in which, the blocked arm was opened and considered to be the novel arm. The animal was allowed to explore all three arms for 5 min and the number and time of entries into each arm were recorded. The experimental environment was kept quiet, under dim light, and each of the arms was cleaned with 70% ethanol between trials.

Notably, a mouse was excluded from analysis if it died during experiments or the performance exceeded the given maximum duration.

### Preparation of EVs and RVG-EVs

Lamp2b-RVG plasmids were transfected into the HEK293T cell line to produce EVs fused with RVG. Briefly, cells were seeded and 24 h later were transfected; 6 h after transfection, cells were washed with PBS and incubated in fresh culture medium (without EVs). The culture supernatant was collected for EV isolation 48 h later. To purify the EVs, we used differential ultracentrifugation as described previously [20]. Briefly, the culture medium was centrifuged at 300 g at 4°C for 10 min to remove detached cells. The supernatant was collected and centrifuged at 2,000 g at 4°C for 10 min to remove cell debris, and the next supernatant was collected and centrifuged at 10,000 g at 4°C for 30 min to remove apoptotic bodies. Then, the supernatant was collected and centrifuged at 100,000 g at 4°C for 70 min to obtain the rough EVs. Finally, the EVs were spun down by ultracentrifugation of the supernatant at 100,000 g for 70 min at 4°C (Beckman 70.1 T1 Ultracentrifuge Rotator), washed in ice-cold filtered PBS, and resuspended in 100 μL ice-cold filtered PBS after centrifugation again. These EV samples are used immediately or stored at −80°C.

### Loading of MiR-100-5p Antagomir into EVs

To load miR-100-5p antagomir into EVs, antagomir (5 nmol, with/without fluorescein [FAM] labelling) was suspended with EVs (800 μg/200 μL), and the final volume was adjusted to 200 µl using the electroporation buffer. Electroporation of the EV-antagomir mixture was carried out using a pulse width of 20 ms and voltages (200 V) a number of pulses (10) following the manufacturer’s protocol. The mixture was then incubated for 30 min at 4°C. To remove unbound antagomir residues, samples were washed with PBS, and after high-speed centrifugation (100,000 g, 70 min), the pellet was resuspended in PBS buffer.

### Characterization of EVs

For conventional characterization of the samples, EVs were purified by differential centrifugation and then characterized by nanoparticle tracking analysis (NTA), transmission electron microscopy (TEM), and Western blot, following the protocols that we established. NTA was used to analyze particle sizes and numbers using the NanoSight LM10 instrument (Malvern Instruments Ltd., Malvern, UK). The microstructure was characterized by TEM, and TEM images were captured by Servicebio Biotechnology Co., Ltd (Wuhan, China). Western blot analysis was applied to assess the protein expression of specific markers to identify EVs (TSG101, Proteintech, 28283-1-AP, rabbit, 1:1000; CD81, Proteintech, 66866-1-LG, mouse, 1:1000; and negative protein Calnexin, Proteintech, 10427-2-AP, rabbit, 1:1000).

### Measurement of EV Uptake* in Vitro* and* in Vivo*

To determine the *in vivo* distribution of modified EVs, modified EVs (EVs-Antagomir^FAM^ and RVG-EVs-Antagomir^FAM^) were administered intranasally to neonatal mice at 12 h post-HI. One hour or 6 h after intranasal infusion, FAM fluorescence signals in the dissected organs (brain, heart, liver, spleen, lung, and kidney) were captured using an IVIS® spectrum *in vivo* imaging system. *In vitro*, primary neurons and PC12 cells were incubated with the modified EVs at 37°C for 6 h, and stained with immunofluorescent microtubule-associated protein 2 (Abcam, ab32454, rabbit, 1:200). The results were observed under a fluorescence microscope (Olympus).

### Statistical Analyses

For each experiment, the minimum sample size was calculated by power and sample size calculators (https://powerandsamplesize.com/Calculators/) from the expected effect size based on preliminary experiments. All graphs were created using GraphPad Prism 8.0.1, and statistics images were analyzed using SPSS Statistics 26 and ImageJ and Fiji software. Significant outliers were calculated using SPSS 26.0 and were excluded from the analysis. Normality tests (Shapiro-Wilk test) and homogeneity of variance were applied to all data before choosing statistical tests. Unpaired Student′s t-test (two-tailed) was used to assess the difference between two independent groups. Data with normal distributions from multiple groups were compared by one-way analysis of variance (ANOVA) with Bonferroni (homoscedasticity) or Dunnett's (heterogeneity of variance) *post hoc* test. Data with skewed distributions from multiple groups were tested using the nonparametric rank-sum test (Wilcoxon test). A repeated measure two-way analysis of variance (ANONA) was used for group comparisons of miR-100-5p expression over time, with “days” as the within-subject factor and “groups” as the between-subject factor. Daily miR-100-5p levels were evaluated using Student′s t-test (two-tailed). Unless otherwise indicated, all data for continuous variables are presented as the mean ± standard deviation (SD) and significance is determined as *P <0.05, **P <0.01, and ***P <0.001. Parallel experiments were repeated more than three times in each group.

## Results

### Knockdown of MiR-100-5p Alleviates Infarct Area and Enhances Behavioral Recovery in HI Mice

**Fig. 1:**
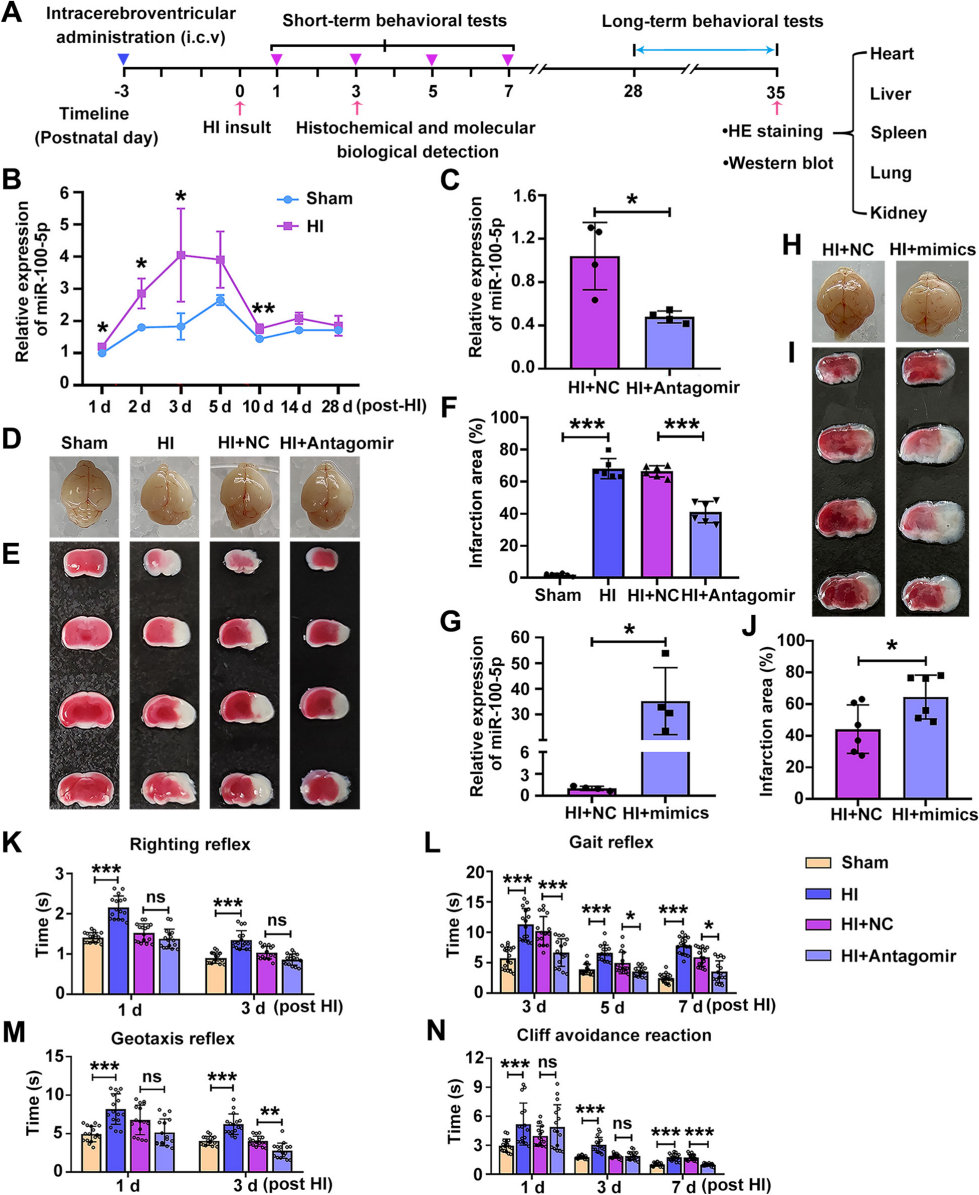
Knockdown of miR-100-5p alleviates cerebral infarction and short-term behavioral impairment in HI mice. **A** Experimental design and timeline for the modeling method, behavior, histology, and molecular biology experiments on mice treated with miR-100-5p antagomir or mimics. **B** qRT-PCR showing MiR-100-5p expression changes over development in the ipsilateral cortex of the Sham and HI groups (*n =* 4). *P <0.05, **P <0.01, Student′s *t-*test. **C** qRT-PCR showing the relative level of miR-100-5p in the ipsilateral cortex of HI mice with miR-100-5p antagomir treatment at 3 days after HI insult (*n =* 4). *P <0.05, Student′s *t-*test. **D** Representative brain images from each group at 3 days post-HI with miR-100-5p antagomir or negative control treatment. **E** Representative brain slices stained by TTC. **F** Analysis of the infarct area in lesion areas in each group (*n =* 6 per group). ***P <0.001, one-way ANOVA with Dunnett's *post-hoc* test. **G** qRT-PCR showing the relative levels of miR-100-5p in the ipsilateral cortex of HI mice with miR-100-5p mimic treatment at 12 h after HI insult (*n =* 4). *P <0.05, Student′s *t-*test. **H** Brain images from a representative mouse from each group at 12 h after HI insult with miR-100-5p mimic or negative control treatment. **I** Representative samples stained with TTC after administration of miR-100-5p mimics or negative control treatment. **J** Analysis of the infarct area in each group (*n =* 6 per group). *P <0.05, Student′s *t-*test. **K-N** Short-term neurobehavioral outcome evaluated *via* the righting reflex, geotaxis reflex, gait reflex, and cliff avoidance reaction, carried out on days 1, 3, 5, and 7 (*n =* 16 per group). *P <0.05, **P <0.01, ***P <0.001, Wilcoxon test with Bonferroni correction in **K**, **L**, and **N**; ***P <0.001, Wilcoxon test with Bonferroni correction at day 1 in **M**, and **P <0.01, ***P <0.001, one-way ANOVA with Dunnett's *post-hoc* test at day 3 in **M**. All data are represented as the mean ± SD.

Our laboratory has been engaged in research investigating the role of microRNAs in neuropathology for some time. During our search for microRNAs linked to HI conditions, we showed that miR-100-5p varied independently in its expression over developmental time in both the Sham and HI groups (Fig. 1B), while its expression significantly increased in the ipsilateral cortex after HI insult, and it was most pronounced on the third-day post-HI (Figs 1B and S1A) compared with the Sham group at the same time point. To validate whether miR-100-5p plays an important role in the pathological processes of HI, we intervened in miR-100-5p expression *in vivo*, and carried out cerebral injury-related behavioral, molecular biological, immunofluorescence, and histological assays (see the flow chart in Fig. 1A). Firstly, we repressed miR-100-5p expression by intraventricular (i.c.v.) injection of miR-100-5p antagomir (50, 100, or 150 pmol) at PND4 and applied 2,3,5‐triphenyltetrazolium chloride (TTC) staining at 3 days post-HI. The results revealed that administration of 150 pmol miR-100-5p antagomir significantly improved brain edema and infarction area of HI mice (Fig. S1C-E). Hence, we used this dose for subsequent experiments. As shown in Figure 1D-F, a clear edematous infarction was evident within the ipsilateral brain at 3 days after HI insult. Compared with the control antagomir-treated mice, the expression of miR-100-5p decreased by 54% at 3 days post-HI (Fig. 1C). MiR-100-5p antagomir treatment (i.c.v.) reduced the HI-induced brain edema as compared with the control antagomir group (Fig. 1D). TTC staining showed a marked restoration of brain infarction areas in the miR-100-5p antagomir treatment group in comparison with the control group (Fig. 1E and F). We next assessed whether miR-100-5p overexpression affects brain edema and infarction following HI (Fig. S1B). Firstly, we determined the effect of miR-100-5p levels on infarct size at 3 days post-HI by TTC staining. Interestingly, administration of miR-100-5p mimics did not have a pronounced effect on brain edema and infarct area at 3 days after HI (Fig. S1F-H), we speculate that this lack of correlation could be due to a ceiling effect. Thus, we next examined brain damage at 12 h after HI by evaluating the infarct area *via* TTC staining. Treatment with miR-100-5p mimics markedly increased the expression of mature miR-100-5p at 12 h after HI insult when compared to the negative control (Fig. 1G). HI mice treated with MiR-100-5p mimics had a significant increase in infarct area as compared to HI + NC at 12 h after the HI insult (Fig. 1H-J).

Neurobehavioral performance was the first parameter used to evaluate recovery. To assess the impact of miR-100-5p on neurological function after HI brain damage, we carried out the behavioral tests, the righting reflex, geotaxis reflex, gait reflex, cliff avoidance reaction, OFT, and Y-maze, in the Sham, HI, HI + NC, and HI + miR-100-5p antagomir groups. In the righting reflex test, animals suffering from an HI insult exhibited a longer latency at 1 and 3 days post-HI compared to the sham group, while HI + miR-100-5p antagomir mice displayed a trend for reduced reflex latency at 1 and 3 days post-HI injury, but the difference from the HI + NC group was not statistically significant (Fig. 1K). In the geotaxis reflex test, results analogous to those of the righting reflex were found. Compared with the sham group, longer reaction times were recorded at both time points in the HI group. However, performance was improved in HI + miR-100-5p antagomir mice by 3 days post-HI (Fig. 1M). Besides, HI mice displayed a similarly increased latency at all time points for the gait reflex and the cliff avoidance test, while miR-100-5p antagomir mice were protected from developing this deficit, with significantly reduced reaction latency at all time points in the gait reflex test (Fig. 1L), but only at 7 days post-HI in the cliff avoidance test (Fig. 1N). Collectively, these results demonstrate that treatment with miR-100-5p antagomir after HI improved the short-term neurobehavioral performance of mice as assessed by the leading tests in reflex and sensorimotor development.

**Fig. 2:**
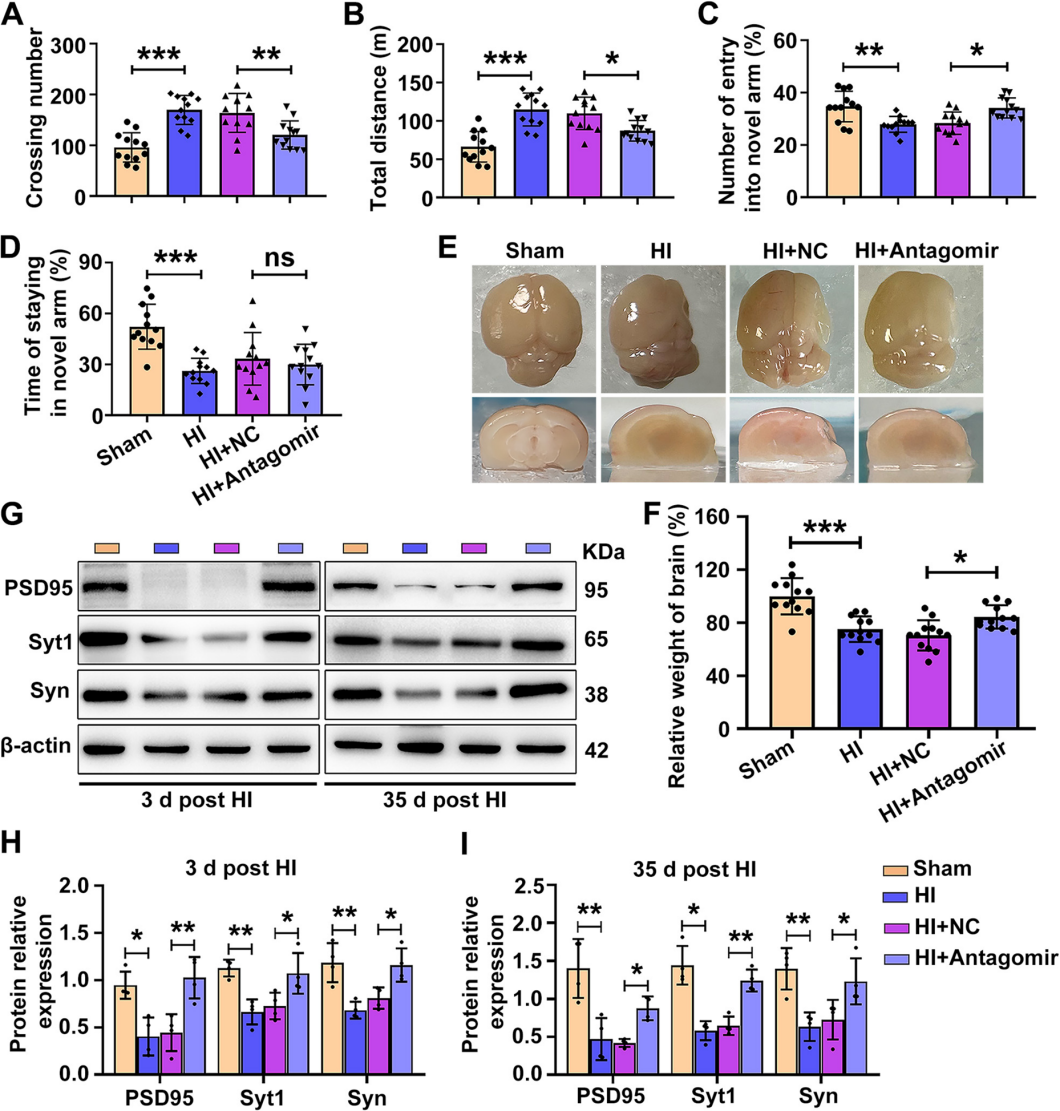
Knockdown of miR-100-5p improves long-term behavioral recovery and the expression of synapse-associated proteins in HI mice. **A-D** Long-term neurobehavioral outcome evaluated via the open field test (OFT) and Y-maze carried out on days 28-35 (*n =* 12 per group). **A** Effect of miR-100-5p antagomir on the number of crossings in the OFT. **P <0.01, ***P <0.001, one-way ANOVA with Bonferroni correction. **B** The effect of miR-100-5p antagomir on the total distance traveled in the OFT. *P <0.05, ***P <0.001, one-way ANOVA with Bonferroni correction. **C**, **D** Percentage of entries into the novel and other arms and times in the Y-maze test. *P <0.05, **P <0.01, ***P <0.001, one-way ANOVA with Bonferroni correction. **E** Representative images of brain atrophy at 35 days after HI insult. **F** Relative weights of brains at 35 days post-HI in the Sham, HI, HI+miR-100-5p NC, and HI+miR-100-5p antagomir groups (*n =* 12 per group). *P <0.05, ***P <0.001, one-way ANOVA, Bonferroni corrected. **G** Representative images of western blots for measuring protein expression levels of PSD95, Syt1, and Syn at days 3 and 35 post-HI. **H** Measurement of PSD95 (*P <0.05, **P <0.01, Wilcoxon test), Syt1, and Syn expression (*P <0.05, **P <0.01, one-way ANOVA, Bonferroni corrected) at day 3 post-HI *via* ImageJ. **I** Measurement of PSD95 (*P <0.05, **P <0.01, one-way ANOVA with Bonferroni correction), Syt1, and Syn expression (*P <0.05, **P <0.01, Wilcoxon test) at day 35 post-HI (*n =* 4). All values represent the mean ± SD.

We next evaluated long-term behavioral outcomes and whether miR-100-5p antagomir treatment can mitigate the impaired motor capacity and learning and memory associated with HI, *via* the OFT and Y-maze, respectively, at 28 days following the HI insult. The OFT can be used to assess the spontaneous locomotion, exploratory behavior, and tension of mice in new environments. (Figs S2A and 2A, B). The numbers of crossings and total distance traveled by HI-insult mice increased as previously reported [21–23], while miR-100-5p antagomir administration significantly decreased the crossing number and total distance traveled compared with the HI + NC group (Fig. 2A, B). The Y-maze is mainly used to study spatial working memory in rodents. In Y maze experiments (Fig. S2B), the number of mice entering the novel arm and residence times in the novel arm were lower in the HI group than in the Sham group, while only the entries of mice in the HI + miR-100-5p antagomir group into the novel arms were increased as compared with those in the HI + NC group (Fig. 2C, D). Collectively, these results showed that treatment with miR-100-5p antagomir promotes recovery from HI-induced motor and memory deficits. We excluded the possibility that miR-100-5p antagomir administration caused systemic toxicity because exogenous miR-100-5p antagomir did not induce pathological changes in the brain, heart, liver, spleen, lung, or kidney (Fig. S3C). At the same time, survival and weight were recorded, and the results indicated that 75% of animals survived to termination in the four groups and slight weight loss (not significantly different) was recorded in HI mice compared with the Sham group at collection (Fig. S3A, B).

### MiR-100-5p Knockdown Ameliorates Long-term Brain Atrophy and Restores Synapse-associated Protein Expression after HI Injury

Compared to the Sham group, the brains of the mice in the HI group exhibited marked atrophy and liquefaction, as indicated by a clear tissue defect area and a reduction in brain weight (Fig. 2E, F). The administration of miR-100-5p antagomir partially suppressed the atrophy (Fig. 2E, F). Western blot analyses revealed a decrease in the expression of postsynaptic density protein 95 (PSD95), Synaptotagmin-1 (Syt1), and Synaptophysin (Syn) at 3 and 35 days after HI insult compared with the Sham group (Fig. 2G-I), while PSD95, Syt1, and Syn expression was significantly restored at 3 and 35 days after miR-100-5p antagomir treatment (Fig. 2G-I). The above results show that miR-100-5p antagomir treatment decreases brain atrophy, as well as restoring the expression of synaptic proteins following HI injury.

### Knockdown of MiR-100-5p Promotes Neuronal Survival in HI Mice

**Fig. 3:**
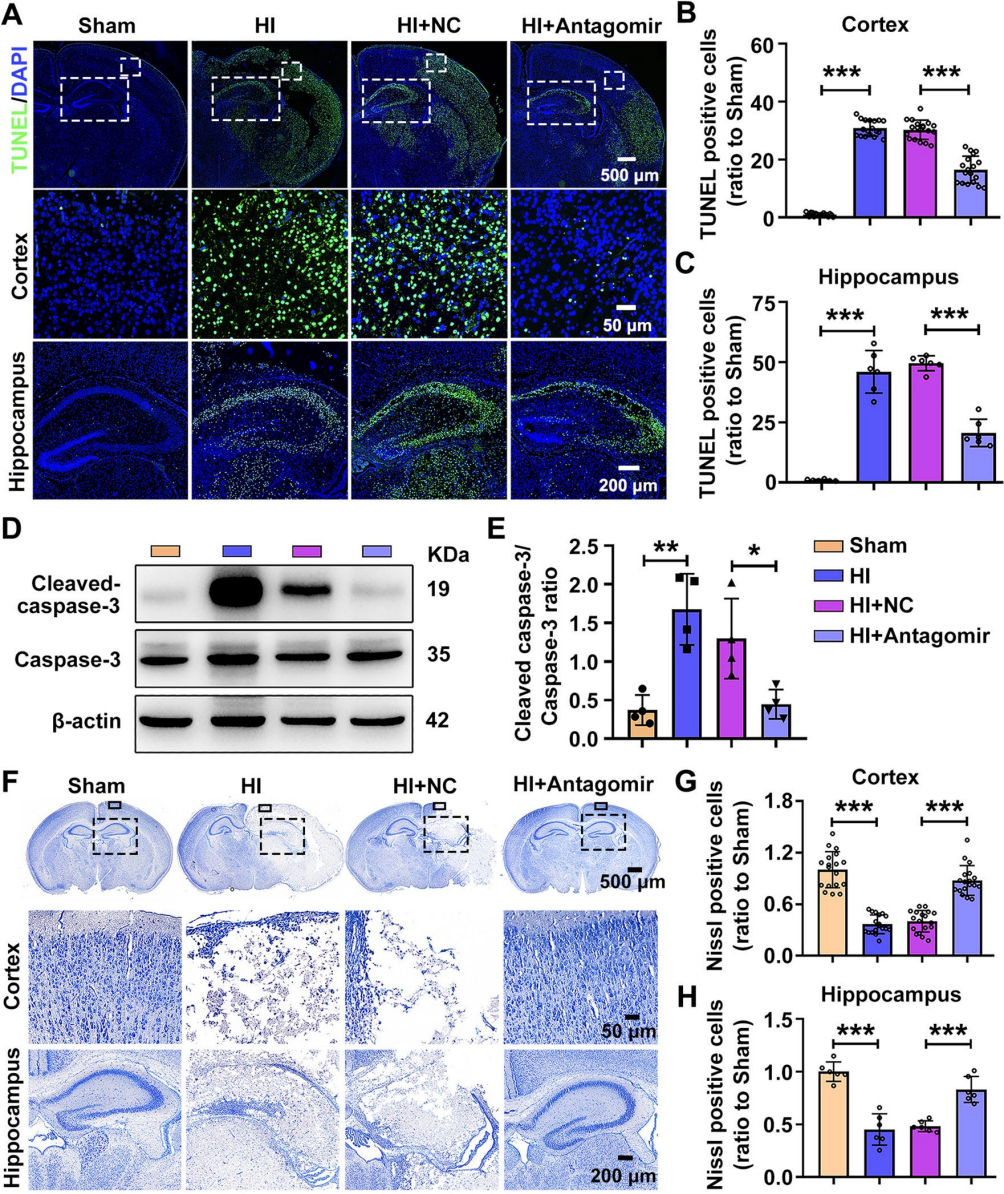
Knockdown of miR-100-5p alleviates neuronal loss in HI mice. **A** Representative TUNEL staining of the brain in each group at day 3 post-HI. Scale bars, 500 μm in **A**, upper; 50 μm in **A**, middle; 200 μm in **A**, lower. **B** Numbers of TUNEL-positive cells in the cortex of each group at day 3 post-HI (*n =* 18 per group from 3 mice). ***P <0.001, one-way ANOVA with *post hoc* Dunnett's correction. **C** Numbers of TUNEL-positive cells in the hippocampus of each group at day 3 post-HI (*n =* 6 per group from 3 mice). ***P <0.001, one-way ANOVA with Dunnett's correction. **D** Western blots of cleaved caspase-3 and caspase-3 within the ipsilateral cortex at day 3 post-HI. **E** The ratios of protein levels of cleaved caspase-3 and caspase-3 (*n =* 4 per group). *P <0.05, **P <0.01, one-way ANOVA followed by Bonferroni *post hoc* test. **F** Nissl staining shows the number of neurons in the ipsilateral cortex and hippocampus of each group. Low-magnification (upper) scale bar, 500 μm; high-magnification (middle), 50 μm; and 200 μm (lower). **G** Numbers of intact neurons (Nissl staining) in cortex (*n =* 18 per group from 3 mice). ***P <0.001, one-way ANOVA followed by Dunnett's *post hoc* test. **H** Numbers of intact neurons (Nissl staining) in the hippocampus (*n =* 6 per group from 3 mice). ***P <0.001, one-way ANOVA with Bonferroni correction. Values represent the mean ± SD.

Neuronal survival was assessed by terminal-deoxynucleotidyl transferase mediated nick end labeling (TUNEL) staining. The results revealed that exposure to HI led to an increase in the number of apoptotic cells compared with the Sham group, while miR-100-5p antagomir treatment significantly reduced apoptotic cell numbers in the cortex and hippocampus, as expected (Fig. 3A-C). At the same time, we examined the changes in the levels of the apoptosis-related proteins cleaved-caspase3 and caspase3. The cleaved-caspase-3/caspase-3 ratio in the ipsilateral cortex increased after HI and decreased after miR-100-5p antagomir administration compared with the HI + NC group (Fig. 3D, E). Stereological evaluation of Nissl-stained coronal sections was used to quantify neuronal loss. In our study, brain sections were collected at 3 days post-HI. Histological evaluation revealed that the Sham group had round nuclei and integral Nissl bodies, whereas the Nissl bodies in the HI group exhibited marked nuclear deviation, and were pathologically changed into particles (Fig. 3F). Meanwhile, the number of Nissl bodies decreased in the HI group (Fig. 3G, H). However, miR-100-5p antagomir treatment greatly rescued the situation, including the changes in quantity and modality (Fig. 3G, H). These data showed that miR-100-5p antagomir effectively reduces HI-induced neuronal death.

A study has shown that the HI model develops intravascular coagulation [24], further leading to long-term dysfunction of blood flow restoration. Therefore, we examined whether miR-100-5p could affect cerebral vascular occlusion and the expression of vascular endothelial growth factor (VEGF) after HI insult. Following the methods described in previous studies [24], the FITC-dextran tracer was administered intracardially 72 h after HI insult, and frozen brain tissue sections were collected at 2 min after intracardial injection for FITC fluorescence measurement; this can serve as an indicator of cerebral vascular patency to a certain extent. We observed that, compared to the sham group, there was extensive vascular obstruction in the hippocampus and cerebral cortex of the injured hemisphere after HI injury, and inhibition of miR-100-5p expression mitigated the vascular obstruction in the injured brain regions compared with the negative control group (Fig. S4A-C). Angiogenetic factors, including VEGF, play an important role in neovascularization, thereby increasing the cerebrovascular density and improving collateral circulation [25]. Thus, we examined the expression levels of VEGF in the injured cortical tissue. The results revealed that, compared to the sham group, the expression of VEGF decreased in the cortical tissue after HI. Crucially, inhibiting miR-100-5p expression alleviated this reduction in VEGF expression compared to the control groups (Fig. S4D-E). These findings suggest that inhibiting miR-100-5p expression can, to a certain extent, ameliorate the decrease in cerebral blood flow and angiogenesis induced by HI injury.

### Knockdown of MiR-100-5p Alleviates Neuroinflammation in HI Mice

**Fig. 4:**
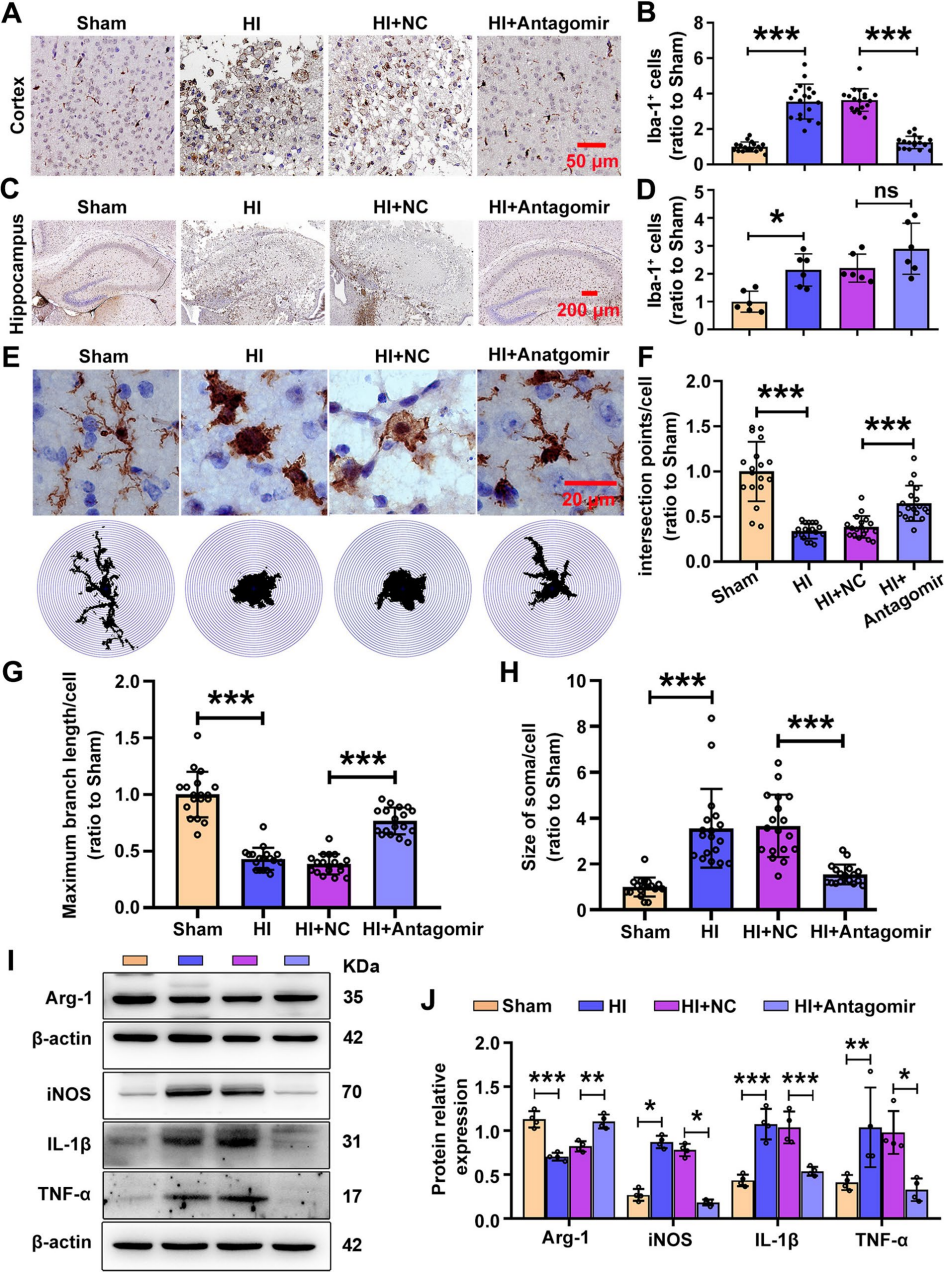
MiR-100-5p knockdown impedes HI-induced microglial activation after HI injury. **A** Representative image of Iba-1 staining in the ipsilateral cortex at day 3 post-HI. Scale bar, 50 μm. **B** Analysis of Iba-1 positive cells within the ipsilateral cortex at day 3 post-HI (*n =* 18 per group from 3 mice). ***P <0.001, one-way ANOVA followed by Dunnett's *post hoc* test. **C** Representative staining of Iba-1 in the ipsilateral hippocampus at day 3 post-HI. Scale bar, 200 μm. **D** Analysis of Iba-1 positive cells in the ipsilateral hippocampus at day 3 post-HI (*n =* 6 per group from 3 mice). *P <0.05, one-way ANOVA followed by Bonferroni correction. **E**, Paraffin section (10 μm) with Iba-1 staining to characterize microglial morphology at day 3 after HI injury. Upper, representative images of microglia (scale bar, 20 μm). Lower, outlines of single microglia using Fiji software (binary image). **F** Sholl analysis of intersections to quantify the complexity of Iba-1-stained microglia; ratios of intersection numbers within a radius of 300 pixels (*n =* 18 per group from 3 mice). ***P <0.001, one-way ANOVA followed by Dunnett's *post hoc* test. **G** The ratio to sham of maximum branch length (*n =* 18 per group from 3 mice). ***P <0.001, one-way ANOVA with Bonferroni correction. **H** The ratio to sham of soma size (*n =* 18 per group from 3 mice). ***P <0.001, one-way ANOVA with Bonferroni’s *post ho*c test. **I** Western blots of levels of Arg-1, iNOS, IL-1β, and TNF-α in the ipsilateral cortex at day 3 post-HI. **J** Levels of Arg-1, iNOS, IL-1β, and TNF-α (*n =* 4 per group). *P <0.05, **P <0.01, ***P <0.001, one-way ANOVA with Bonferroni correction. All values represent the mean ± SD.

Neuronal damage typically promotes an inflammatory response in the form of microglial activation. The activation of microglia was assessed by examining the level of Iba-1 protein within the ipsilateral cortex at 3 days after HI *via* immunohistochemistry. The results reveled a significant increase in the number of Iba-1^+^ cells in the core region of the cortical infarct of HI mice at 3 days post-HI injury, compared with the Shan group (Fig. 4A, B). The administration of MiR-100-5p antagomir significantly impeded microglial activation by reducing the number of Iba-1^+^ cells (Fig. 4A, B). We found a similar phenomenon in the hippocampus, but miR-100-5p antagomir treatment failed to reduce the number of microglia (Fig. 4C, D). Next, we assessed the morphology of microglia by Sholl analysis. Significant morphological changes in individual microglia were observed in both the ipsilateral cortex and hippocampus. The results showed a shift toward a more reactive morphology after HI insult, exhibited a remarkable decrease in the number of ramifications and maximum branch length in microglial (Fig. 4E-G). Conversely, microglia developed a larger soma and a “bushy” appearance or became rounded/amoeboid macrophage-like cells post-HI (Fig. 4H). The above morphology improved significantly after miR-100-5p antagomir treatment (Fig. 4E-H). Consistent signs of histological injury were observed in the cortex and hippocampus after neonatal HI, and severe injury resulted in failure to harvest the hippocampus, therefore, we focused the subsequent analysis on the molecular changes in the cortex.

To assess the extent to which the decreased miR-100-5p expression influenced the expression of inflammatory genes, we measured the protein levels of three cytokines associated with pro-inflammation [inducible nitric oxide synthase (iNOS), interleukin-1β (IL-1β), and tumor necrosis factor α (TNF-α)], as well as arginase-1 (Arg-1), which is associated with anti-inflammation. As Figure 4I and J show, a significant fold increase in the pro-inflammatory cytokines and a decrease in the anti-inflammatory cytokine occurred in the lesioned cortex of mice subjected to HI as compared to the control. However, miR-100-5p antagomir significantly mitigated the expression of the pro-inflammatory cytokines iNOS, IL-1β, and TNF-α, and increased the expression of the anti-inflammatory cytokine Arg-1 in the lesioned cortex as compared to NC-treated HI mice (Fig. 4I, J). Interestingly, miR-100-5p mimics directly boosted the production of inflammatory factors (iNOS and TNF-α) in BV2 cells. Moreover, it further aggravated the increased expression of inflammation-related proteins (iNOS and TNF-α) caused by oxygen and glucose deprivation/re-oxygenation (OGD/R) exposure (Fig. S9B-D).

### Engineered RVG-EV-Antagomir *via* Intranasal Delivery Targets the Injured Region

**Fig. 5:**
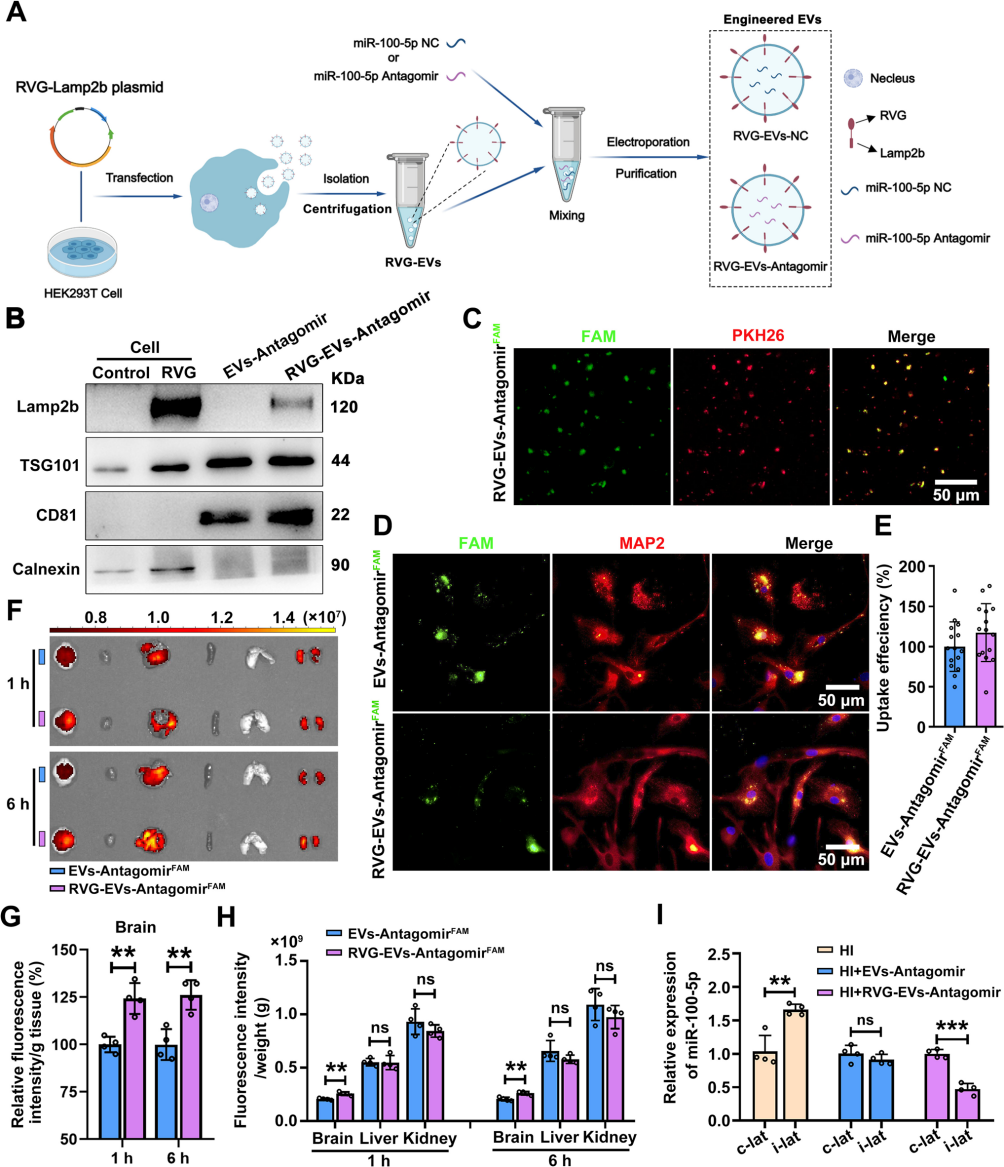
Physical characterization of the engineered EVs and their distribution *in vivo* and *in vitro*.** A** Schematic of the production and harvest of RVG-EVs for targeted delivery of miR-100-5p antagomir (created with MedPeer: www.medpeer.cn). **B** Western blot analysis of EVs-Antagomir and RVG-EV-Antagomir for specific identified EVs proteins, as well as common EVs (TSG101, CD81) and non-EVs (Calnexin) protein markers. **C** Representative images of RVG-EVs-Antagomir (FAM-labeled antagomir loaded into PKH26-labeled RVG-EVs by electroporation). **D** The uptake of EVs-Antagomir^FAM^ and RVG-EVs-Antagomir^FAM^ *in vitro* was analyzed by incubation with primary neurons. **E** Quantification of internalization of primary neuronal exosome signals in the RVG-EVs-Antagomir^FAM^ group and EVs-Antagomir^FAM^ group. 5 fields of each cell culture slide (n = 3) are quantified. **F** Fluorescence images of EVs-Antagomir^FAM^ and RVG-EVs-Antagomir^FAM^ in major organs captured by the IVIS Spectrum system at 1 h and 6 h after intranasal infusion of EVs-Antagomir^FAM^ and RVG-EVs-Antagomir^FAM^ (*n =* 4). **G** Quantification of brain fluorescence signals at 1 h and 6 h after intranasal infusion of EVs-Antagomir^FAM^ and RVG-EVs-Antagomir^FAM^ (*n =* 4). **P <0.01, Student′s *t-*test. **H** Fluorescence intensity per gram of tissue in brain, liver, and kidney at 1 h and 6 h after intranasal infusion of EVs-Antagomir^FAM^ and RVG-EVs-Antagomir^FAM^ (*n =* 4). **P <0.01, Student′s *t-*test. **I** qRT-PCR showing the relative miR-100-5p levels in the contralateral and ipsilateral cortex of HI mice at 48 h following EVs-Antagomir and RVG-EVs-Antagomir treatment (*n =* 4). **P <0.01, ***P <0.001, Student′s *t-*test. All values represent the mean ± SD.

EVs derived from various bacteria or mammalian cells have higher delivery efficiency and better biocompatibility, which renders them attractive as miRNA nanocarriers. Previous studies have shown that peptides derived from RVG can be displayed on the surface of particles to enhance neuronal targeting [26]. In fact, it has been reported that RVG confers neuronal targeting capacity due to the binding to acetylcholine receptors in neurons [27]. Since the delivery of therapeutic miRNAs to brain cancer is limited by the BBB, we generated RVG-EVs loaded with miR-100-5p antagomir (RVG-EVs-Antagomir) that target the injured region of the brain. In brief, we transfected HEK293T cells with plasmids that encode the RVG-Lamp2b fusion protein. Next, RVG-EVs were isolated from the supernatant of cultured cells using gradient centrifugation, and the miR-100-5p antagomir was loaded into RVG-EVs through electroporation followed by RVG-EVs-Antagomir purification (Fig. 5A). EVs-Antagomir was generated in a similar way, it is just that EVs were isolated from the HEK293T cell culture supernatant (Fig. S5A). Then, basic characterization of EVs-Antagomir and RVG-EVs-Antagomir was performed. The engineered EVs that we generated exhibited a uniform physical appearance, having a cup-shaped morphology with diameters varying from 100 to 150 nm, as accurately measured through nanoparticle tracking analysis and electron microscopy (Fig. S5B, C). EVs identity was further confirmed by western blotting using exosome-specific antibodies. As depicted in Figure 5B, Lamp2b was expressed in HEK293T cells and successfully integrated into the RVG-EVs derived from these cells. And exosome-specific markers (TSG101 and CD81) were positive, while Calnexin was negative. In order to verify that miR-100-5p antagomir was effectively loaded into RVG-EVs, we labeled RVG-EVs-Antagomir^FAM^ using the red fluorescent molecules of PKH26, and fluorescence images confirmed the presence of the miR-100-5p antagomir^FAM^ (green) inside the RVG-EVs (Fig. 5C).

The efficiency of the intracellular delivery of RVG-EVs-Antagomir nanoparticles was evaluated in neurons *in vitro*. The FAM-labeled antagomir for miR-100-5p was used to prepare the EVs-Antagomir^FAM^ and RVG-EVs-Antagomir^FAM^ nanoparticles. The EVs-Antagomir^FAM^ and RVG-EVs-Antagomir^FAM^ were added to primary neurons and PC12 cells and the intracellular uptake of miR-100-5p antagomir after 6 h was measured by immunofluorescence (Fig. S5D). The results showed that the EVs-Antagomir^FAM^ and RVG-EVs-Antagomir^FAM^ were taken up by primary neurons (Fig. 5D). However, there was no significant difference in the uptake capacity of both EVs by primary neurons (Fig. 5E), but there was a difference in PC12 cells *in vitro* (Fig. S6A, B). MiR-100-5p expression was effectively decreased in PC12 cells after incubation with EVs-NC, EVs-Antagomir, RVG-EVs-NC, and RVG-EVs-Antagomir (Fig. S6C). These results confirm the successful conjugation of RVG to EVs, which in turn increased the cellular uptake and targeting of exosomes to neurons. In addition, we also tested the cytotoxicity in PC12 cells by CCK8 assays (Fig. S5D). These showed that EVs, RVG-EVs, and the engineered EVs we generated did not display increasing cytotoxicity as the concentration increased (Fig. S7A-D). Furthermore, treatment with EVs-Antagomir (300 pmol/60 μg) and RVG-EVs-Antagomir (200 pmol/40 μg and 300 pmol/60 μg) reversed the decreased viability of PC12 cells under OGD conditions (Fig. S8A, B). On the contrary, our study found that mir-100-5p mimics did not have any influence on the viability of PC12 cells under normal conditions. However, when PC12 cells were subjected to OGD/R stimulation, the presence of mir-100-5p mimics exacerbated the death process of these cells (Fig. S9A).

To determine the distribution in various tissues of EVs administered intranasally, the EVs-Antagomir^FAM^ and RVG-EVs-Antagomir^FAM^ were resuspended in PBS and intranasally administered to mice (each mouse was treated with 40 μg EVs, including 200 pmol miR-100-5p antagomir^FAM^) at 12 h after HI insult. At 1 and 6 h after administration, different organs were dissected and analyzed using the IVIS® spectrum *in vivo* imaging system (Fig. S10A). The results showed that the uptake of EVs by the brain was enhanced by ~25% at 1 and 6 h after intranasal administration through RVG conjugation compared with the uptake of naïve EVs *in vivo* (Fig. 5F, G); it is interesting to note that the fluorescence in the brain of the RVG-EVs-Antagomir^FAM^ group was mainly distributed in the right injured brain regions of HI-mice, while it was relatively uniformly distributed in the EVs-Antagomir^FAM^ group. However, there was no significant difference in the fluorescence intensity of other organs (heart, liver, spleen, lung, and kidney) between the indicated groups (Fig. 5F, H). These data suggested that under HI pathological conditions, RVG-EVs-Antagomir^FAM^ has a great ability to target the brain by penetrating the BBB. Moreover, quantitative real-time PCR (qRT-PCR) revealed that miR-100-5p expression was significantly decreased in the ipsilateral cortex of HI-mice compared with the contralateral cortex, and the efficiency in the RVG-EVs-Antagomir^FAM^-injected group was markedly superior to the EVs-Antagomir^FAM^-injected group (Figs 5I and S10B, C). These data suggested that RVG-EVs-Antagomir selectively targeted the lesion area compared with unmodified EVs-Antagomir.

### Intranasal Delivery of RVG-EVs-Antagomir Attenuates Brain Damage

**Fig. 6:**
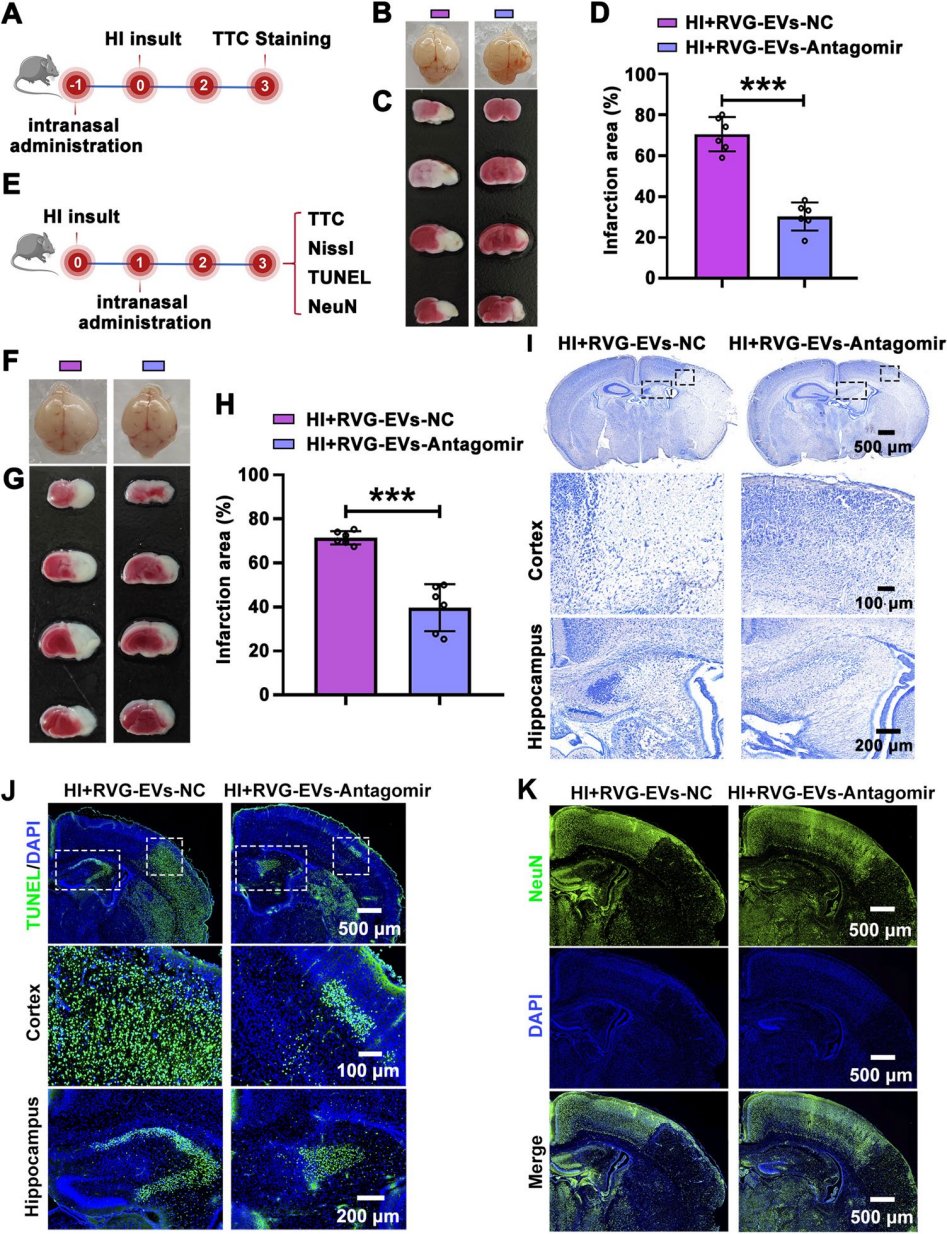
Intranasal delivery of the RVG-EVs-Antagomir attenuates brain damage post-HI injury. **A** The design and timeline of *in vivo* experiments for **B-D.** RVG-EVs-Antagomir or RVG-EVs-NC is administered *via* intranasal instillation at 24 h pre-HI injury. The pups on PND7 have right common carotid artery ligation, followed by the induction of hypoxia (9.7% O_2_+90.3% N_2_ for 1 h). TTC staining is applied on day 3 post-HI insult. **B** Representative images of the brain acquired from each group on day 3 post-HI through the intranasal delivery of RVG-EVs-Antagomir or RVG-EVs-NC. **C** Representative brain slices stained with TTC staining to quantify the infarct area. **D** Analysis of the infarct area in each group (*n =* 6 per group). ***P <0.001, Student′s *t-*test. **E** The design and timeline of *in vivo* experiments for **F-K**. The pups on PND7 undergo right common carotid artery ligation, and hypoxia is induced, then RVG-EVs-Antagomir or RVG-EVs-NC is intranasally administered 24 h after HI injury. TTC, Nissl, TUNEL, and NeuN staining were applied at day 3 post-HI insult. **F** Representative brain images acquired from each group at day 3 post-HI after intranasal delivery of RVG-EVs-Antagomir or RVG-EVs-NC. **G** Representative brain slices with TTC staining to quantify infarct area. **H** Analysis of the infarct area in each group (*n =* 6 per group). ***P <0.001, Student′s *t-*test. **I** Representative brain sections with Nissl staining to measure neuronal survival. Scale bars, 500 μm (upper), 100 μm (middle), 200 μm (lower). **J** Representative images of TUNEL staining. Scale bars, 500 μm (upper), 100 μm (middle), 200 μm (lower). **K** Immunofluorescence images of NeuN in the ipsilateral cortex of HI mice following RVG-EVs-Antagomir treatment. Scale bar, 500 μm. All values represent the mean ± SD.

Subsequently, we investigated the impact of RVG-EVs-Antagomir on the recovery from brain damage in HI mice. PND6 mice were given the RVG-EVs-NC/Antagomir by intranasal administration followed by HI insult at 24 h. TTC staining was applied at 3 days post-HI (Fig. 6A). The results showed that the RVG-EVs-Antagomir effectively prevented the cerebral edema and infarction caused by HI (Fig. 6B-D). Taking into account clinical relevance, we also evaluated the impact of administering RVG-EVs-Antagomir intranasally at 24 h post-HI injury (Fig. 6E). The TTC staining results demonstrated that RVG-EVs-Antagomir treatment at 24 h post-HI insult also relieved the HI-induced brain edema and infarction compared to RVG-EVs-NC (Fig. 6F-H). Meanwhile, we found that EVs and RVG-EVs nanoparticles alone had no mitigating effect on HI-induced cerebral edema and infarction (Fig. S11).

Nissl and neuronal nuclear antigen (NeuN) staining results showed that RVG-EVs-Antagomir intervention reversed the HI-induced neuronal loss in the ipsilateral cortex and hippocampus (Figs 6I, K and S12A, B, E, F). TUNEL staining indicated that RVG-EVs-Antagomir treatment reduced the HI-induced apoptosis (Figs 6J and S12C, D). Taken together, pre- and post-HI administration of RVG-EVs-Antagomir *via* nasal delivery had similar neuro-protective effects on acute brain injury following HI injury.

### Ppp3ca is a Direct Target of MiR-100-5p and may Alleviate HI-Induced Brain Damage *via* an Anti-apoptotic Effect in Neonatal Mice

**Fig. 7:**
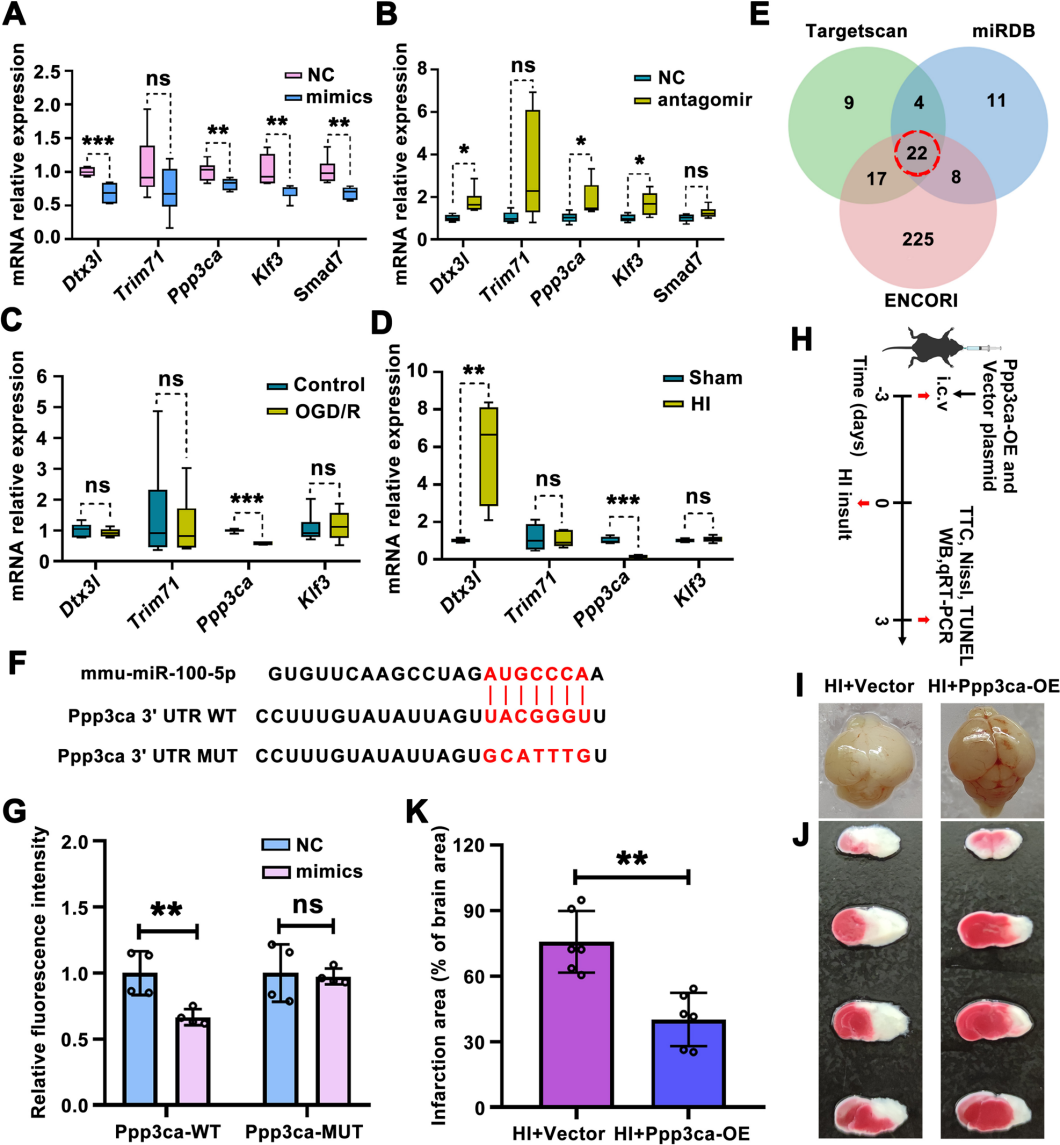
*Ppp3ca* is a direct target of miR-100-5p and a mediator of its adverse effects on neuronal apoptosis. **A** The relative expression of *Dtx3l*, *Trim71*, *Ppp3ca*, *Klf3*, and *Samd7* in PC12 cells (qRT-PCR) transfected with the miR-100-5p mimics or negative control (*n =* 4). **P <0.01, ***P <0.001, Student′s *t-*test. **B** The relative expression of *Dtx3l*, *Trim71*, *Ppp3ca*, *Klf3*, and *Samd7* in PC12 cells (qRT-PCR) transfected with the miR-100-5p antagomir or negative control (*n =* 4). **P <0.01, ***P <0.001, Student′s *t-*test. **C** The relative expression of *Dtx3l*, *Trim71*, *Ppp3ca*, and *Klf3* in PC12 cells after OGD/R exposure (*n =* 4). ***P <0.001, Student′s *t-*test. **D** The relative expression of *Dtx3l*, *Trim71*, *Ppp3ca*, and *Klf3* in the ipsilateral cortex of Sham and HI groups (*n =* 4). **P <0.01, ***P <0.001, Student′s *t-*test. **E** Venn diagram of miR-100-5p target genes screened from TargetScan, miRDB, and ENCORI databases. **F** Construction of the *Ppp3ca* dual-luciferase reporter gene and its potential sequence to which miR-100-5p binds. **G** The relative luciferase activity of the miR-100-5p mimics and NC groups respectively expressing *Ppp3ca*-WT and *Ppp3ca*-MUT (*n =* 4). **P <0.01, Student′s *t-*test. **H** Experimental design and timeline for investigating the role of *Ppp3ca* in HI injury mediated by miR-100-5p. *Ppp3ca* overexpression plasmids (10 μg per mouse) or equivalent vector plasmids are administered to the mice *via* unilateral stereotaxic microinjection into the lateral ventricle on day 3 prior to HI insult. 3 days after HI, the mice are sacrificed for examination of infarction, apoptosis, and inflammation. **I** Representative images of the brain collected from each group on day 3 post-HI. **J** Representative brain slices with TTC staining to quantify infarct area. **K** Analysis of the infarct area in each group (*n =* 6 per group). **P <0.01, Student′s *t-*test. Values represent the mean ± SD.

**Fig. 8:**
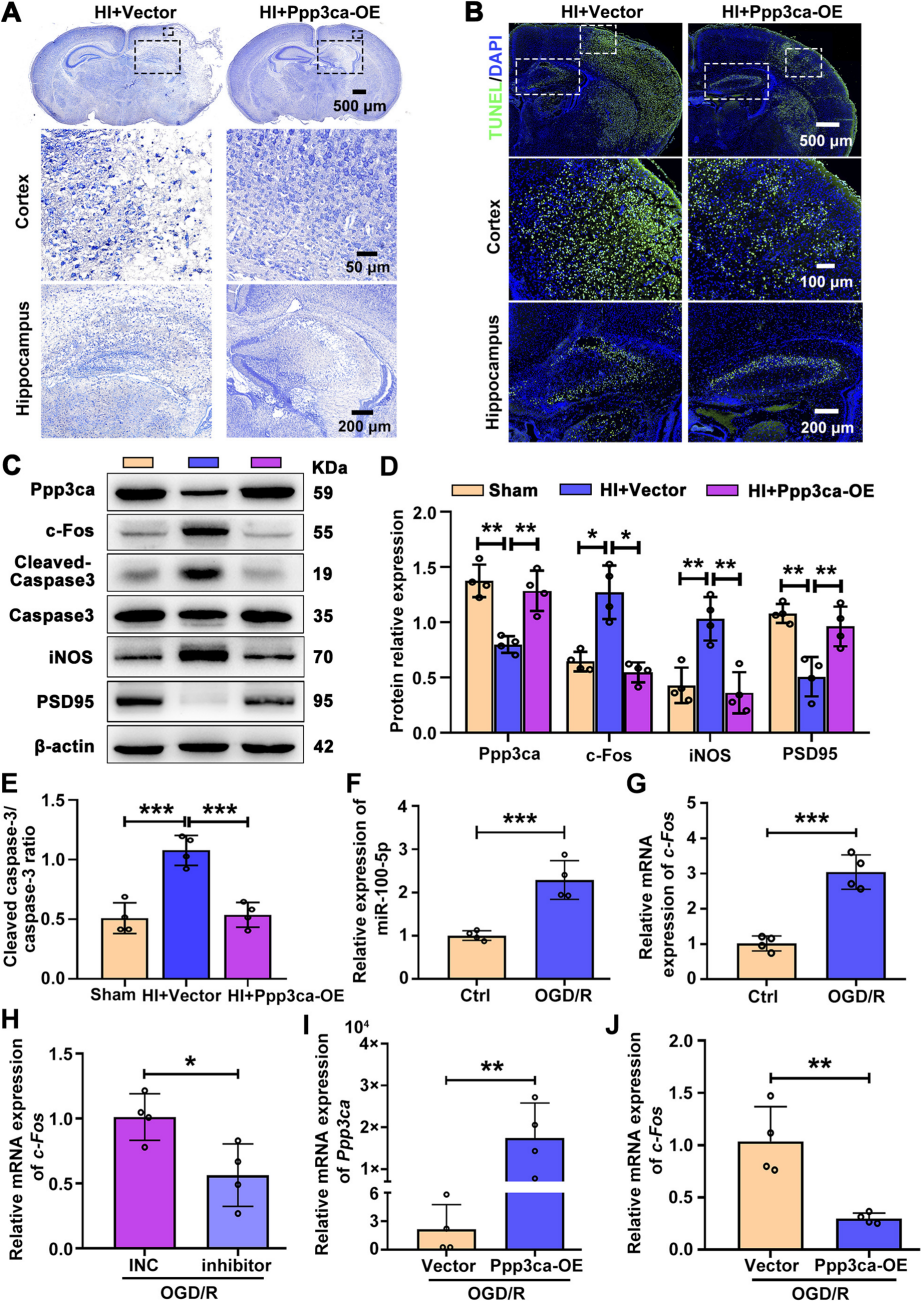
Overexpression of Ppp3ca alleviates brain injury in HI mice. **A** Representative sample Nissl stained to determine neuronal survival. Scale bars, 500 μm (upper), 50 μm (middle), 200 μm (lower). **B** Representative images of TUNEL staining. Scale bars, 500 μm (upper), 100 μm (middle), 200 μm (lower). **C** Western blot of the relative protein levels of Ppp3ca, c-Fos, cleaved-caspase3, total caspase3, iNOS, and PSD95 in the ipsilateral cortex of different groups. **D** Relative protein levels of Ppp3ca, c-Fos, iNOS, and PSD95 in different groups (*n =* 4). Data are normalized to β-actin. *P <0.05, **P <0.01, one-way ANOVA followed by Bonferroni post-hoc test. **E** The ratio of protein levels of cleaved caspase3 and caspase3 (*n =* 4 per group). ***P <0.001, one-way ANOVA followed by Bonferroni *post-hoc* test. **F** Relative expression of miR-100-5p in PC12 cells after OGD/R exposure (*n =* 4). ***P <0.001, Student′s *t-*test. **G** Relative expression of c-Fos in PC12 cells after OGD/R exposure (*n =* 4). ***P <0.001, Student′s *t-*test. **H** Relative expression of c-Fos in PC12 cells with OGD/R exposure after miR-100-5p antagomir or NC transfection (*n =* 4). *P <0.05, Student′s *t-*test. **I** Relative expression of Ppp3ca in PC12 cells with OGD/R exposure after transfection with Ppp3ca overexpression plasmids or vector plasmids (*n =* 4). **P <0.01, Student′s *t-*test. **J** Relative expression of c-Fos in PC12 cells with OGD/R exposure after transfection with Ppp3ca overexpression plasmids or vector plasmids (*n =* 4). **P <0.01, Student′s *t-*test. All values represent the mean ± SD.

To further investigate the mechanism underlying the promotion of neuronal survival by RVG-EVs-Antagomir, we screened for potential target genes of mir-100-5p. Considering the increased expression of miR-100-5p and the neuronal apoptosis phenotype observed in the models of HI brain damage (Figs 1 and 3), we focused on miR-100-5p target genes associated with neuronal apoptosis. Firstly, potential target genes of miR-100-5p were defined by the intersection predicted using three databases (TargetScanMouse 7.2, miRDB, and ENCORI) (Fig. 7E). Next, we chose deltex E3 ubiquitin ligase 3l (*Dtx3l*), tripartite motif containing 71 (*Trim71*), protein phosphatase 3 catalytic subunit alpha (*Ppp3ca*), KLF transcription factor 3 (*Klf3*), and sterile alpha motif domain containing 7 (*Samd7*) from the intersection and verified which was the true target gene involved with miR-100-5p biological activity. *In vitro*, miR-100-5p mimics, miR-100-5p antagomir, and their negative control were incubated with PC12 cells for 48 h followed by qRT-PCR. In PC12 cells, treatment with miR-100-5p mimics decreased the mRNA levels of *Dtx3l*, *Ppp3ca*, *Klf3*, and *Samd7*, as compared to mimics NC (Fig. 7A), while miR-100-5p antagomir treatment increased *Dtx31*, *Ppp3ca,* and *Klf3* expression, as compared to miR-100-5p antagomir NC (Fig. 7B). In addition, OGD/R exposure significantly decreased the *Ppp3ca* mRNA level in PC12 cells (Fig. 7C). In line with this result, HI also decreased the *Ppp3ca* mRNA level in the lesioned cortex (Fig. 7D). We focused on the genes having a negative relationship with the change in miR-100-5p expression in the lesioned cortex (Fig. S1A) and OGD-exposed cells (Fig. 8F). Among them, *Ppp3ca* was selected for further exploration, and the binding and regulatory relationships with miR-100-5p were verified. Prediction analysis showed that miR-100-5p may bind within the 1405-1411 location of the *Ppp3ca* 3′ untranslated region (Fig. 7F). Therefore, we confirmed the direct binding between *Ppp3ca* and miR-100-5p by performing a dual-luciferase assay, the results of which showed that miR-100-5p mimics significantly decreased the fluorescence activity (Fig. 7G), and that this effect was abrogated when the binding site was mutated (Fig. 7G).

Then we further evaluated the specific role of Ppp3ca in HI brain damage (Fig. 7H). Ppp3ca-overexpressing plasmid was introduced into HI-injured mice to enhance Ppp3ca expression. Reassuringly, the up-regulated expression of Ppp3ca partially blocked the HI-induced edema (Fig. 7I) and infarct area (Fig. 7J, K). However, overexpression of miR-100-5p partially antagonized the neuroprotective effect of Ppp3ca overexpression, as the protective effect of Ppp3ca on HI-induced cerebral edema, infarction (Fig. S13A-C), and early abnormal neural reflex development (Fig. S13D, E) was abolished by overexpression of miR-10-5p. Nissl staining showed that Ppp3ca overexpression reversed the HI-reduced number of Nissl bodies in the ipsilateral cortex and hippocampus (Figs 8A and S14A, B). Likewise, increased Ppp3ca relieved the apoptosis caused by HI insult, as shown by TUNEL staining (Figs 8B and S14C, D).

Furthermore, we confirmed that Ppp3ca overexpression also significantly reduced the cleaved-caspase3/caspase3 ratio (Fig. 8C, E) and the expression of the pro-inflammatory cytokine iNOS (Fig. 8C, D). Inversely, it restored the PSD95 expression (Fig. 8C, D). Previous studies have reported that c-Fos expression is negatively regulated by Ppp3ca [28]. As a marker of neuronal activity, the c-Fos level was assessed *in vivo* and *in vitro*. The results showed that the c-Fos mRNA (Fig. S15B) and protein (Fig. 8C, D) levels were significantly elevated in the ipsilateral cortex following the HI insult. However, overexpression Ppp3ca (Fig. S15A) reversed the HI-induced elevation of c-Fos (Figs 8C, D and S15). Uniformly, *in vivo*, the miR-100-5p level (Fig. 8F) and *c-Fos* mRNA level (Fig. 8G) were up-regulated in PC12 cells following OGD/R exposure. And inhibition of mir-100-5p expression (Fig. 8H) or overexpression of *Ppp3ca* (Fig. 8I, J) both reversed the elevation of *c-Fos* caused by HI injury.

To sum up, these data confirmed that Ppp3ca is a direct miR-100-5p target *in vivo* and may be a key mediator of the effects of miR-100-5p on neuronal apoptosis after HI insult.

## Discussion

In the present study, we present miR-100-5p as a potential therapeutic target for clinical intervention in HI brain injury. Initially, we confirmed that miR-100-5p was significantly upregulated in the lesioned cortex of HI mice. Subsequent assessment of the efficacy of a miR-100-5p antagonist in mitigating HI insult revealed that the administration of miR-100-5p antagomir led to a marked improvement in behavioral impairments, attenuation of neuronal damage, and reduction in microglial activation, as compared to the negative control group. We further demonstrated the utility of engineered EVs incorporating RVG and miR-100-5p antagomir (termed RVG-EVs-Antagomir), which effectively downregulated the miR-100-5p levels in the injured brain region following a single intranasal dose. Importantly, both 24 h pre- and post-HI administration of RVG-EVs-Antagomir evoked comparable therapeutic responses to HI insult. Mechanistic studies identified Ppp3ca as a novel target of miR-100-5p. By inhibiting c-Fos expression, Ppp3ca suppressed neuronal apoptosis subsequent to HI insult. In conclusion, we have established a non-invasive method utilizing engineered EVs for the successful delivery of a miR-100-5p antagonist to the brain and demonstrated that downregulating miR-100-5p expression significantly enhances functional recovery post-HI insult by targeting Ppp3ca to suppress neuronal apoptosis.

The present study has potential application value for the clinical treatment of HI brain injury, primarily manifested in the following aspects: firstly, HI injury is a major cause of neurological dysfunction in both neonates and adults. In this study, we demonstrated that downregulating miR-100-5p expression significantly promotes functional recovery following HI brain injury, which offers a new therapeutic target for patients with such injury, potentially improving their prognosis and quality of life. Notably, our study showed that the therapeutic window for targeting mir-100-5p can be extended to 24 h after HI injury, which is important for patients who cannot receive timely treatment. Secondly, traditionally, delivering therapeutic agents to the brain presents numerous challenges, including the hindrance of the BBB and the risks associated with invasive treatment modalities. We successfully achieved non-invasive delivery of a miR-100-5p antagonist using engineered EVs as carriers, providing a novel and safe strategy for the treatment of brain diseases. Thirdly, we further elucidated the molecular mechanism by which miR-100-5p inhibits neuronal apoptosis *via* targeting Ppp3ca. This not only deepens our understanding of the pathophysiological processes following HI brain injury but also provides a scientific basis for the future development of more precise and targeted therapeutic strategies. In summary, this study not only provides new ideas and methods for the treatment of HI brain injury but also opens up new avenues for in-depth research and the clinical treatment of neurological diseases.

Our research findings show that miR-100-5p promotes the pathological progression of HI brain injury, and inhibiting its expression alleviates the brain damage induced by HI insult. However, we cannot exclude the possibility that miR-100-5p has distinct biological effects in other models of brain injury. In the CNS, miR-100-5p released from apoptotic cortical neurons can activate endogenous TLR7/8 to trigger further neuronal apoptosis [7]. Cao et al. reported that miR-100-5p promotes the autophagy response through binding to mTOR protein, thereby inhibiting apoptosis and delaying the progression of cerebral infarction (CI) [29]. They established the mouse model of CI by middle cerebral artery occlusion (MCAO). In the experimental design, the authors did not examine the expression changes of miR-100-5p in the ischemic cortex of MCAO mice compared to Sham mice and, thus we cannot definitively ascertain the alterations in miR-100-5p levels within the brain tissue after CI. It is not rigorous to conclude that the level of miR-100-5p in the brain of animals with CI is decreased merely based on the speculation derived from finding reduced mir-100-5p levels in the blood of patients with acute CI from the GEO datasets. However, in recent research, Xin et al. found that miR-100-5p can aggravate microglial activation and neuronal damage after stroke *via* activating the TLR7/NF-κB pathway [30]. Interestingly, they used the same animal model. Thus, the inconsistent roles of miR-100-5p reported in stroke models and neonatal HI models may stem from a multitude of factors, including differences in the models themselves, timing and modes of intervention, interactive networks, and experimental conditions and manipulations, as well as biological variability and individual differences.

The cornerstone enabling technology that underpins our research and scientific breakthroughs was the development of engineered EVs capable of delivering miR-100-5p antagomir into the brains of HI-mice. Due to limitations by the BBB, delivering drugs into the brain has always been a major challenge in the treatment of brain diseases. EVs, which are characterized by low immunogenicity, good natural stability, long half-life, high delivery rate, and the ability to cross the BBB, have become a research hotspot for safe and effective brain drug delivery carriers [31, 32]. However, some problems with natural EVs as a drug delivery system for the brain still need to be solved, such as limited accumulation in disease sites and low therapeutic effects in the brain. The biodistribution pattern of EVs can be advantageously modified by the presentation of surface ligands, including RVG, to augment the accumulation of EVs in disease sites [33]. For example, Haroon et al. coupled rabies virus glycoprotein 29 with the small EVs surface to increase neuron targeting efficiency and treatment for brain injuries including traumatic brain injury and ischemic stroke [26, 34]. EVs can encapsulate proteins, nucleic acid drugs, chemotherapeutic drugs, and traditional Chinese medicine monomers, including curcumin, quercetin, and paclitaxel. Like cell membranes, EVs have a lipid bilayer structure, which can effectively encapsulate hydrophobic and hydrophilic drugs and are especially suitable for the encapsulation of nucleic acid drugs. Accumulating research findings have illustrated that altering the microRNA expression in EVs has therapeutic benefits in mouse models [35–37]. Depending on the nature of the drug, methods such as plasmid transfection [38], saturated solution incubation [39], sonication [40], and electroporation [16] can be used to load drugs into EVs. In this study, we successfully loaded the miR-100-5p antagomir into RVG-modified EVs *via* electroporation. Previous experiments have shown that the RVG-modified nanoparticles rapidly deliver targeting ligands to the infarct site following ischemic stroke [33, 41]. This is consistent with the previous findings that modified RVG-EVs can be used to administer miR-100-5p antagomir to the infarct site through intranasal delivery [33]. We found a significant decrease in infarct volume and apoptotic cells in the RVG-EVs-Antagomir group after HI. Our results just revealed that RVG-EVs-Antagomir can be taken up by primary neurons and PC12 cells, and we did not attempt to quantify its uptake by other cell types, but RVG-EVs-Antagomir seems to be internalized by other cell types as well [41]. Future studies identifying the targeting peptide specific for neural progenitors are still needed.

The underlying mechanism of miR-100‐5p action was also explored in our study and we found that it down-regulates Ppp3ca. Calcineurin A, a catalytic subunit of calcineurin, is encoded by three genes: Ppp3ca, Ppp3cb, or Ppp3cc. Calcineurin is a Ca^2+^-dependent serine/threonine protein phosphatase that is highly expressed in the CNS [42]. It is a heterodimer composed of one catalytic subunit (∼60 kDa, known as calcineurin A) and one regulatory subunit (∼19 kDa, known as calcineurin B). A number of reports have shown that calcineurin is a mediator of astrocytosis in various models of brain insult [43, 44]. For example, active calcineurin in astrocytes abrogates the inflammatory response after TNF-a or endotoxins and markedly enhances neuronal survival [43]. In human and rodent models of neurodegenerative diseases, several regions of the brain show calcineurin A immunoreactivity in neurons [45]. Researchers have confirmed that calcineurin A inhibitors, including FK506 and cyclosporin A, have shown promising therapeutic potential for the treatment of stroke [46, 47], Alzheimer’s disease [48], and Huntington’s disease [49] in recent years. The novel calcineurin A (Ppp3ca) variant associated with epilepsy, exhibits constitutive enzyme activation and the downregulation of protein expression [50]. In immune cells, calcineurin controls the activity of a wide range of transcription factors, including the nuclear factor of activated T cells, nuclear factor-kappa B, c-Fos, and Ets-like protein-1 [28]. Thus, calcineurin A plays a crucial role in T cell activation, cell growth, apoptosis, neuronal depotentiation, and angiogenesis. c-Fos has been reported in several studies to play a role in the modulation of neuronal apoptosis [51, 52]. Here, we found that the level of Ppp3ca was reduced in the lesioned cortex of HI mice and OGD/R-exposed neurons, and was associated with an increased c-Fos level. Overexpression of Ppp3ca can attenuate HI brain damage and OGD/R-induced neuronal apoptosis, and down-regulate c-Fos expression, suggesting that Ppp3ca could potentially play a role in neuronal apoptosis following HI insult *via* regulating c-Fos expression.

While these findings indicate that miR-100-5p is a promising target for therapeutic intervention in HI-induced brain damage, it is important to acknowledge that behavioral tests and immunostaining with limited sample sizes have an increased likelihood of yielding false positive results. In addition, our research only substantiates that, in the short term after HI occurrence, intervening with miR-100-5p antagomir can effectively mitigate acute brain injury and potentially prevent its long-term progression into irreversible permanent damage *via* suppressing inflammation and apoptosis. However, due to the lack of assessment of miR-100-5p antagomir's metabolic half-life *in vivo* and the absence of continuous administration, its direct pharmacological effect on long-term functional recovery following HI remains to be elucidated, highlighting a limitation of the current study. We also cannot exclude the likelihood that this treatment may confer brain protection *via* other underlying mechanisms. In addition, our investigation into the role of Ppp3ca in neuronal apoptosis is preliminary.

## Conclusion

Our study demonstrated the miR-100-5p is increased in the ischemic brain and promotes apoptosis by targeting Ppp3ca. RVG-EVs-Antagomir can be intranasally instilled at 24 h after the onset of HI and still yield beneficial effects. Therefore, the evidence from our data indicates that RVG-EVs-Antagomir can indeed contribute to the recovery of neurological function after cerebral ischemia in neonatal mice and has great potential for therapeutic applications.

## Supplementary information

Supplementary information (PDF 3917 kb)

## Data Availability

All data pertaining to this study are included in the paper or the Supplementary Information. In addition, all relevant data are accessible from the corresponding author upon reasonable request.
