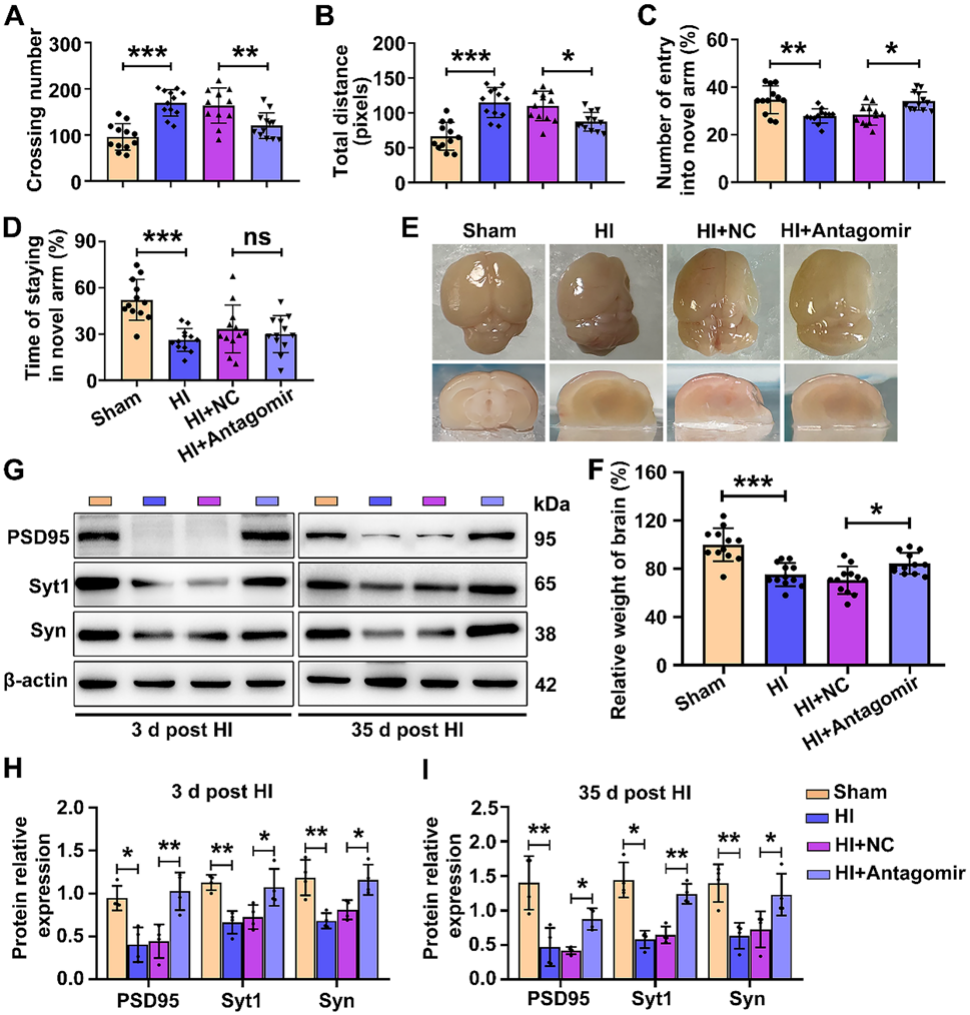# Correction to: Engineered Extracellular Vesicles Loaded with MiR‑100-5p Antagonist Selectively Target the Lesioned Region to Promote Recovery from Brain Damage

**DOI:** 10.1007/s12264-025-01540-y

**Published:** 2025-11-05

**Authors:** Yahong Cheng, Chengcheng Gai, Yijing Zhao, Tingting Li, Yan Song, Qian Luo, Danqing Xin, Zige Jiang, Wenqiang Chen, Dexiang Liu, Zhen Wang

**Affiliations:** 1 Department of Physiology, School of Basic Medical Sciences, Cheeloo College of Medicine, Shandong University, Jinan, 250012 China; 2 Department of Medical Psychology and Ethics, School of Basic Medicine Sciences, Cheeloo College of Medicine, Shandong University, Jinan, 250012 China; 3 Qilu Hospital, Cheeloo College of Medicine, Shandong University, Jinan, 250012 China

**Correction to: Neurosci. Bull.** 10.1007/s12264-025-01376-6

In this article, the unit of total distance in Fig. 2B was misused. In fact, the unit of total distance exported by the system was “pixels”. After correction, the data in Fig. 2B were not changed.

The Fig. 2B should have appeared as shown below: