# Supplementary material for: Engineered Extracellular Vesicles Loaded with MiR-100-5p Antagonist Selectively Target the Lesioned Region to Promote Recovery from Brain Damage
Source: Neurosci Bull. 2025 Apr 1;41(6):1021–40. doi: 10.1007/s12264-025-01376-6 (PMC12158875; doi:10.1007/s12264-025-01376-6)
Supplement: Supplementary file 1 — Supplementary information (PDF 3917 kb) [file 12264_2025_1376_MOESM1_ESM.pdf]

Supplementary Information

Supplementary Materials and Methods

Intracerebroventricular (i.c.v) Injection

We administered intracerebroventricular injections as reported previously [1]. The mouse pups (PND4) were anesthetized using isoflurane and placed on a small animal stereotaxic apparatus (Shenzhen Ward Life Science and Technology Co. Ltd. Shenzhen, China). A small dot was marked on the skin above the skull and the injection site was identified at 2/5 of the distance from the lambda suture to each eye. The needle of 10 μL Hamilton syringe was inserted perpendicularly into a depth of 3 mm through the small dot and 3 μL of miR-100-5p antagomir (50 pmol/μL) or equal amounts of negative control (NC) were slowly administered at an infusion rate of 0.5 μL/min. The syringe was withdrawn 10 min after the injection, and the neonates were allowed to recover from anesthesia on a homeothermic blanket and then placed back into their dams. In addition, miR-100-5p overexpression (miR-100-5p mimics and NC (150 pmol/3 μL)) was induced by i.c.v injection at 3 d before HI modeling. And Ppp3ca overexpression were induced by administration of Ppp3ca-OE plasmid (10 μg/5 μL) by intracranial injection into the right lateral cerebral ventricle of neonatal mice as described previously.

TTC Staining and Infarct Area Evaluation

Three days after HI, mice were overdosed on anesthetic and brains were quickly removed from the skull, and frozen at −20 °C for 20 min after taking photos. Then continuous coronal slices (four slices from each mouse) of the brain tissue (the cerebellum and olfactory bulb were removed) were obtained at 2 mm intervals, stained with 2% TTC (Sigma, St Louis, USA) solution at a constant temperature of 37 °C for 20 min and avoid light. The sections were photographed and the infarcted

areas of the brain were determined by examining TTC-stained slices for the areas that did not stain with TTC using Image J software.

$$\text{Infarct area (\%)}=\frac{\text{contralateral hemisphere areas-heathy areas of ipsilateral hemisphere}}{\text{contralateral hemisphere areas}}\times 100\%$$

TUNEL Staining

The paraffin-embedded brain was cut into 4-μm sections to detect cell death in brain tissues using a TUNEL Cell Apoptosis Detection Kit (Servicebio, Wuhan, China). Briefly, brain slides were deparaffinized, hydrated, treated with proteinase K at 37 °C for 20 min, and incubated with terminal deoxynucleotidyl transferase (TdT) for 1 h in a black humidified box at 37 °C to label apoptotic DNA with UTP-digoxigenin after washing three times with PBS. Paraffin sections were then washed and stained with 4',6-diamidino-2-phenylindole (DAPI) at room temperature for 8 min for nuclear staining. After the sample staining was completed, the tissue samples were washed with PBS for 3 times and anti-fluorescence quenching sealing agent was dropped to seal the slices. Finally, the slides were photographed using a fluorescence microscope.

Nissl Staining

Brain paraffin sections (4 μm in thickness) were stained with Nissl (1% toluidine blue) for 20 min at 60 ℃ after dewaxing and hydrating. After rinsing quickly in distilled water, the sections were differentiated in 95% ethylalcohol for 5~10 seconds (s). After dehydration, the slices were sealed with permount and scanned with a microscope (Olympus vs120). The cells containing Nissl bodies were considered as neurons and counted manually via Image J.

IHC Assays, H&E and IF Staining

Brain tissue paraffin sections (10 μm in thickness) were prepared routinely. The expression of Iba-1 was examined by IHC using BenchMark XT kit (Gene Tech, Shanghai, China). Briefly, sections

were deparaffinized in xylene and then hydrated in graded alcohol of 100%, 95% and 75%. Then antigen retrieval was performed using a microwave oven and quenched for endogenous peroxidases in 3% H_2O_2 for 10 min. After blocking in 1% normal goat serum, the cores were incubated at 4°C overnight with 1:200-diluted primary antibody (Iba-1, Servicebio, GB113502-100). Subsequently, the sections were incubated with corresponding HRP-conjugated secondary antibody for 60 min. The slices were visualized using 3,3'-diaminobenzidine tetrahydrochloride (DAB) and counterstained with hematoxylin, dehydrated and mounted in permount. Stained slides were viewed using an OLYMPUS microscope and images were captured under 40× and 100× magnification. On day 35 after HI, mice were sacrificed and peripheral organ tissues (heart, liver, spleen, lung, kidney) were harvested for hematoxylin and eosin (H&E) staining.

Immunofluorescence (IF) staining was performed as follows. For histological samples, paraffin-embedded 5-μm sections were deparaffinized and rehydrated in successive baths of xylene and ethanol (100%, 95%, 70%, and 50%), followed by heat-induced (95 °C) epitope retrieval in 10 mM sodium citrate buffer (pH = 6.0). After 1 h block with 5% BSA/PBS at room temperature, samples were incubated overnight at 4 °C with primary antibodies diluted in PBS. The next day, sections were washed three times with PBS prior to incubation with secondary antibody diluted in PBS for 60–90 min at room temperature. Then, sections were again washed three times with PBS, labeled with DAPI for nuclear visualization, rinsed with PBS, and mounted in ProLong Gold Antifade Mountant. All primary antibodies were diluted at a 1 to 100 concentration and all secondaries at 1 to 200 concentration unless otherwise specified. The information on antibodies is listed below. Mouse anti-MAP2 (abcam, 254143), Mouse anti-NeuN (abcam, 104224), Rabbit anti-VEGFA (proteintech, 19003-1-AP).

Sholl Analysis

Microglial morphological analysis was performed using FIJI software (Version 2.0) and the Sholl analysis plugin (http://fiji.sc/Sholl Analysis) [2, 3]. For each animal, a minimum of 3 cells was used to perform morphometric analysis per imaging region (incomplete microglia and microglia with processes on the margin were excluded). For Sholl analysis, a line was drawn from the center of the soma extending to the endpoint of the longest branch. The Sholl analysis plugin was used to draw concentric circles starting from 0 pixel followed by 300 pixels successive shells in order to identify the number of intersections along the Sholl radii [4]. Further, default settings of the FIJI Sholl analysis plugin were used (Radius step size = 20 pixels, starting radius = 10 pixels, ending radius = 300 pixels, Sholl Method = linear) and collected for each cell. We also measured the size of cell soma and the longest branch of the cell.

Target-Luciferase Reporter Assay

The miRNA-target luciferase reporter assay was performed as previously described [5]. Dual-luciferase reporter assay was used to confirm whether Ppp3ca is a direct target of miR-100-5p. The wild-type (WT) and 3'-UTR mutant (Mut) of Ppp3ca was amplified and cloned into the pmirGLO luciferase reporter vector (Vazyme, Nanjing, China). The WT and Mut Ppp3ca constructs were co-transfected with either miR-100-5p mimics or Negative control miRNA into the HEK293T cell lines using Lipofectamine 2000. Forty-eight hours following transfection, the luciferase activity was measured using a Dual-Luciferase Reporter Assay Kit (Vazyme) following the manufacturer's protocol. Data are presented as the ratio of Renilla luciferase to firefly luciferase.

Protein Extraction and Western Blot

Brains tissues were harvested from euthanized mice at 3- and 35-day post-HI and total protein was extracted from tissues using RIPA lysis buffer containing a protease inhibitor (PMSF) according

to the manufacturer's instructions. Protein concentrations were determined using BCA Protein Assay Kit (Servicebio, Wuhan, China). Proteins (10~20 μg) from tissue lysates were separated by 12% SDS-PAGE and electrophoretic transferred to PVDF membranes (Millipore). Proteins were treated with purified primary antibodies for overnight at 4°C followed by a horseradish peroxidase-linked secondary anti-rabbit or anti-mouse antibody. Antigen-antibody complexes were visualized by ECL (Millipore). The acquired images were analyzed by ImageJ. Antibodies used and their respective dilutions are as follows: rabbit anti -Calcineurin A (Servicebio, 11669, 1:1000), rabbit anti-beta Actin (Servicebio, 15003, 1:2000), mouse anti-cFos (abcam, 208942, 1:1000), rabbit anti-PSD95 (proteintech, 20665-1-AP, 1:800), rabbit anti-Syt1 (proteintech, 20665-1-AP, 1:5000), rabbit anti-Syn (proteintech, 14511-1-AP, 1:5000), rabbit anti-Arg-1 (proteintech, 16001-1-AP, 1:1000), mouse anti-TNF-α (proteintech, 60291-1-LG, 1:800), rabbit anti-IL-1β (abcam, 9722, 1:800), rabbit anti-Cleaved-caspase-3 (cell signaling technology, 9661S, 1:1000), rabbit anti-Casepase3 (proteintech, 19677-1-AP, 1:1000), rabbit anti-iNOS (proteintech, 18985-1-AP, 1:1000), rabbit anti-VEGFA(proteintech, 19677-1-AP, 1:1000).

qRT-PCR

Total RNA was collected using Trizol (Shenggong, Shanghai, China) from brain tissues according to the manufacturer's recommendations [6]. And the total RNA from cells using rapid cell RNA extraction kit (SPARKeasy, Jinan, China) according to the manufacturer's instructions. cDNA was synthesized using Revert Aid First Strand cDNA Synthesis Kit (TOYOBO, Osaka, Japan). And transcripts were amplified using SYBR green PCR kit (SPARKeasy, Jinan, China) with the ABI7500 (Applied Biosystems) with specific primer sets. Reactions were run in triplicates for each sample and no-template blanks were used as negative controls. Values were normalized to the β-actin (for mRNA)

and U6 snRNA (for miRNA). Primer sequences were as follows:

miR-100-5p: F 5′-AACCCGTAGATCCGAACTTGTG-3′

R 5′-AGTGCAGGGTCCGAGGTATT-3′

Ppp3ca: F 5′-CTGGCTGCGCTAATGAACCA-3′

R 5′-GGTCTGACCATAGGATGTCACAC-3′

Trim71: F 5′-GCTCACGAAGGCTACAGGAG-3′

R 5′-GCCGAGGACCACAACTGAC-3′

Klf3: F 5′-GAGTACCCCTGTCTATGCCG-3′

R 5′-CGACAAGGAAACCATGAGAGG-3′

Dtx3l: F 5′-ACGGGCTCGTTTCTAACTCTG-3′

R 5′-CACTACCCTCCATGCTTTTGAT-3′

Smad7: F 5′-GCATTCCTCGGAAGTCAAGAG-3′

R 5′-CCAGGGGCCAGATAATTCGT-3′

c-Fos: F 5'-AAGTAGTGCAGCCCGGAGTA-3'

R 5'-CCAGTCAAGAGCATCAGCAA-3'

References

[1] Gai C, Xing X, Song Y, Zhao Y, Jiang Z, Cheng Y*, et al.* Up-Regulation of miR-9-5p Inhibits Hypoxia-Ischemia Brain Damage Through the DDIT4-Mediated Autophagy Pathways in Neonatal Mice. Drug Des Devel Ther 2023, 17: 1175-1189.
[2] Ferreira T, Ou Y, Li S, Giniger E, van Meyel DJ. Dendrite architecture organized by transcriptional control of the F-actin nucleator Spire. Development 2014, 141: 650-660.
[3] Ristanović D, Milosević NT, Stulić V. Application of modified Sholl analysis to neuronal dendritic arborization of the cat spinal cord. J Neurosci Methods 2006, 158: 212-218.
[4] Heindl S, Gesierich B, Benakis C, Llovera G, Duering M, Liesz A. Automated Morphological Analysis of Microglia After Stroke. Front Cell Neurosci 2018, 12: 106.
[5] Lei W, Kang W, Nan Y, Lei Z, Zhongdong L, Demin L*, et al.* The Downregulation of miR-200c Promotes Lactate Dehydrogenase A Expression and Non-Small Cell Lung Cancer Progression. Oncol

Res 2018, 26: 1015-1022.
[6] Livak KJ, Schmittgen TD. Analysis of relative gene expression data using real-time quantitative PCR and the 2(-Delta Delta C(T)) Method. Methods 2001, 25: 402-408.

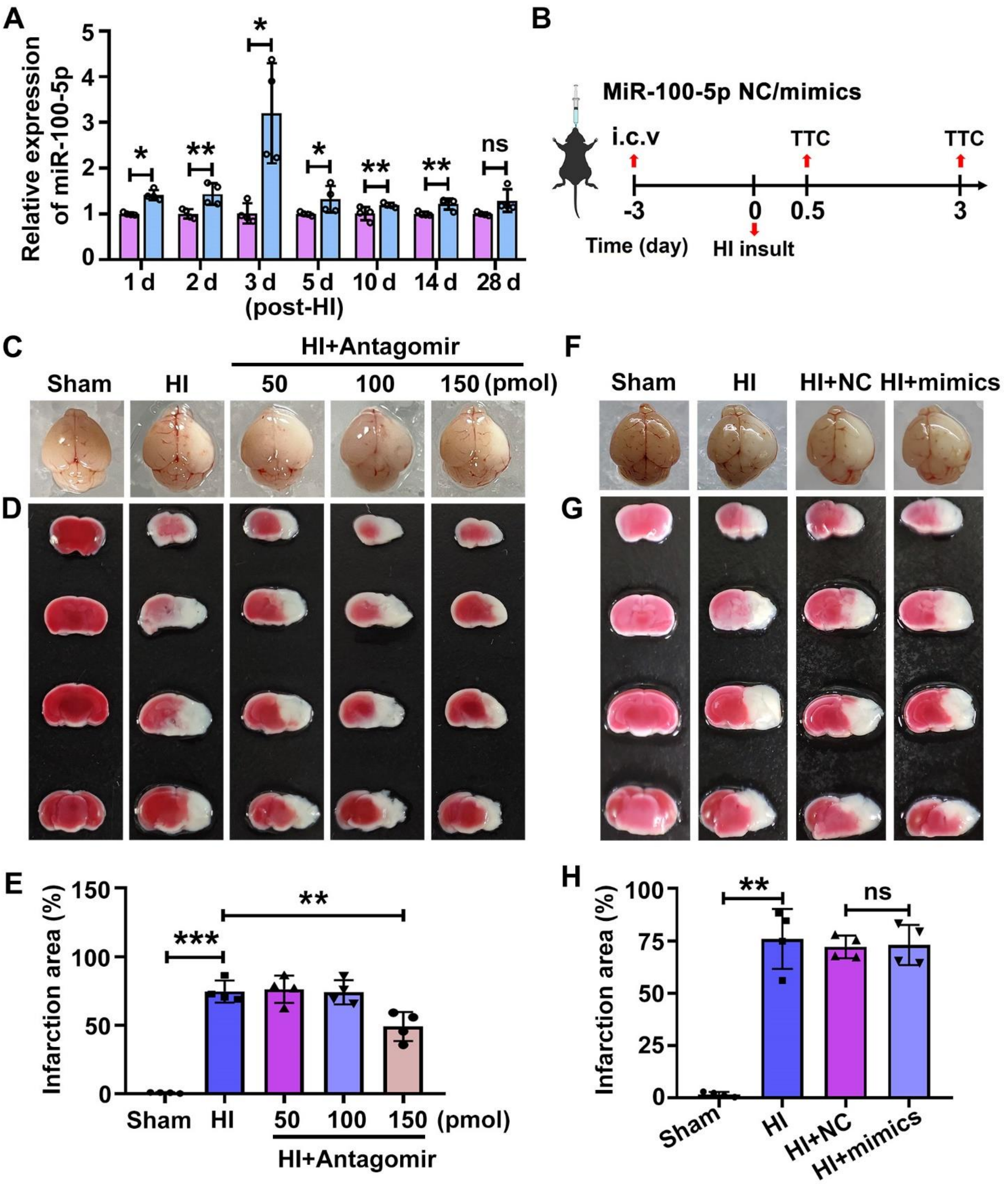

Fig. S1 MiR-100-5p mimics has no effect on cerebral infarction at 3 d after HI. **A** The relative level of miR-100-5p in the ipsilateral cortex of Sham and HI group at different time points were measured with

qRT-PCR (n = 4 per group). * P < 0.05, ** P < 0.01 according to Student′s t-test. **B** Experimental design and timeline for Figure 1G-J and Figure S1F-H. **C** Representative brain edema images were obtained from each group at 3 d post HI via different concentrations of miR-100-5p antagomir treatment. **D** Representative samples stained with TTC. **E** Quantitative analysis of the infarct area in lesion areas in each group (n = 4 per group). ** P < 0.01, *** P < 0.001 according to One-way ANOVA with Bonferroni post-hoc. **F** Representative brain edema images were obtained from each group at 3 d post HI via miR-100-5p mimics or negative control treatment. **G** Representative samples stained with TTC. **H** Quantitative analysis of the infarct area in lesion areas in each group (n = 4 per group). ** P < 0.01 according to One-way ANOVA with Dunnett's post-hoc. All data are represented as mean ± SD.

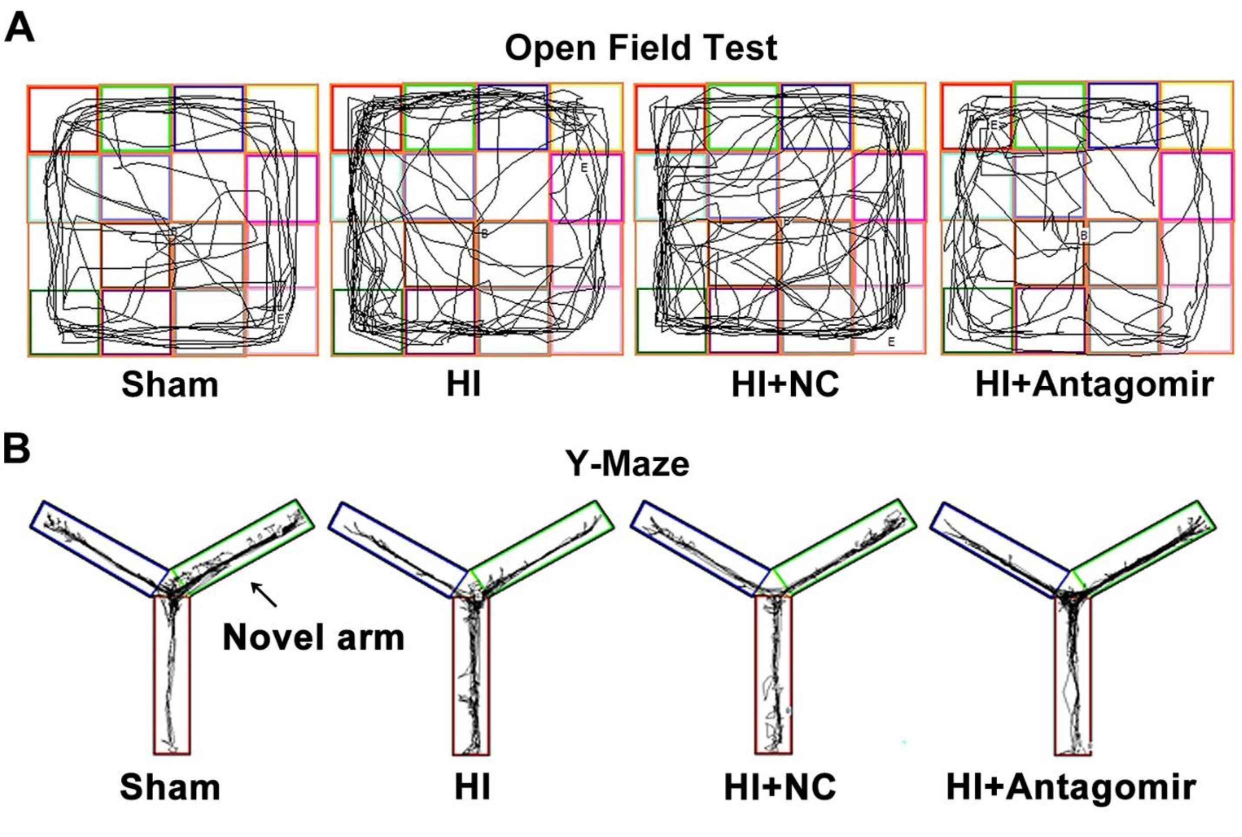

Fig. S2 Long-term behavioral tests trajectories. **A** The representative activity track map of each group in OFT. **B** The representative activity track map of each group in Y-maze.

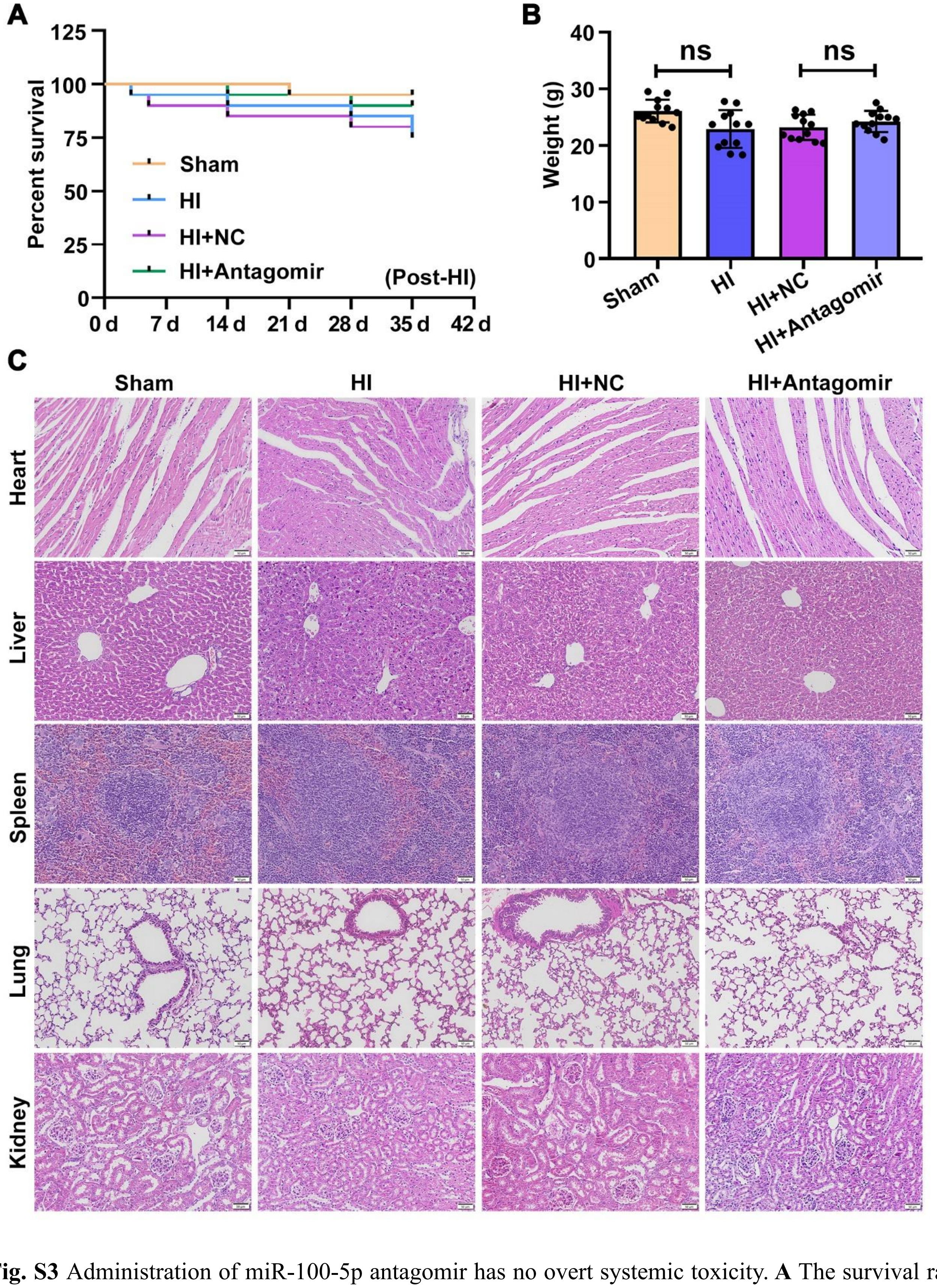

Fig. S3 Administration of miR-100-5p antagomir has no overt systemic toxicity. **A** The survival rate of all groups was calculated at the end of the experiment. **B** The animal weight of all groups was

recorded at 35 d after HI (n = 12 per group). **C** Representative HE staining of main visceral organs in mice. Scale bar=50 μm.

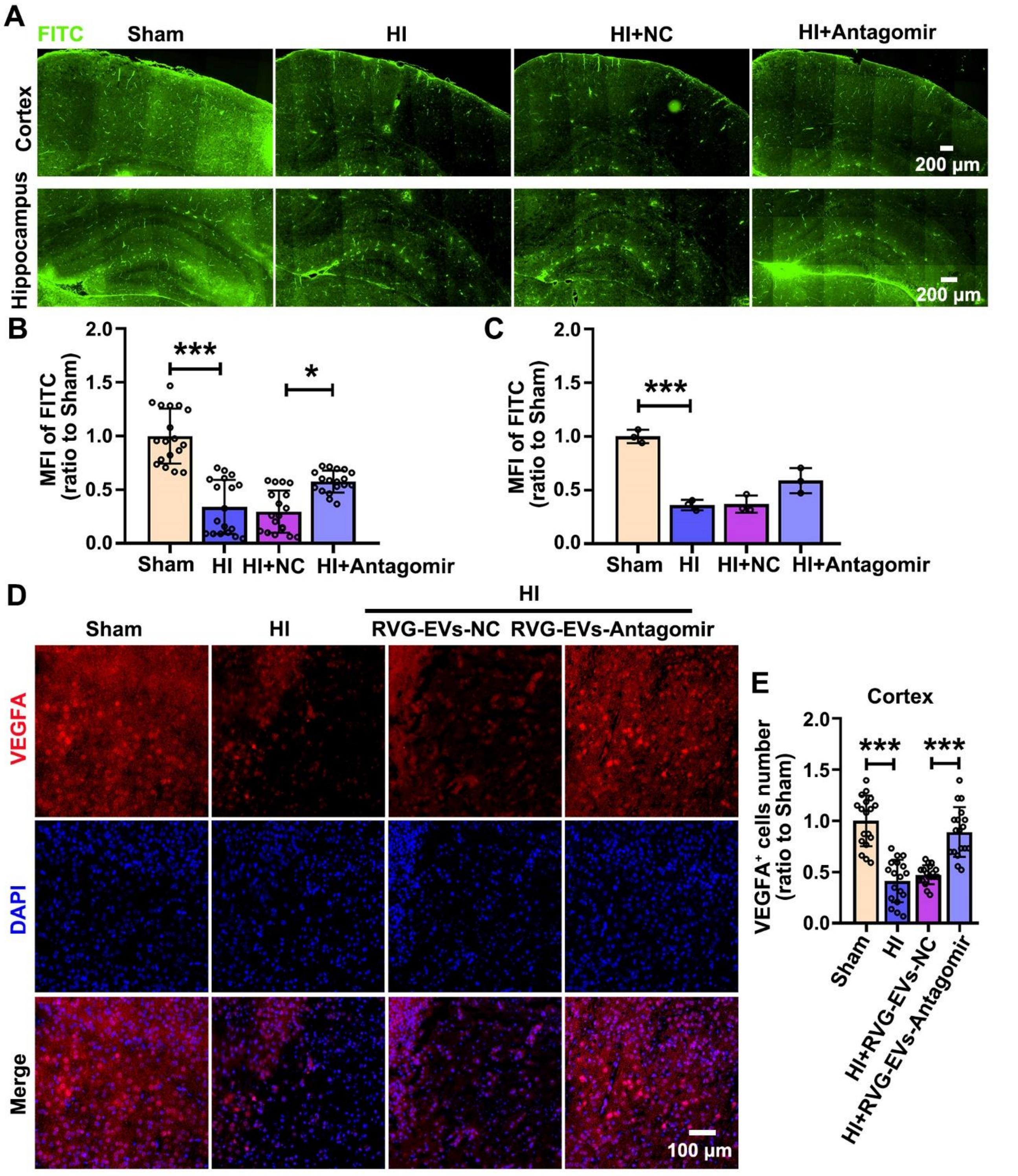

Fig. S4 Knockdown of miR-100-5p alleviates vascular obstruction on the ipsilateral side of the brain in HI mice. **A** Representative fluorescence images of brain by intracardiac injection of FITC-dextran

at 72 hours after HI insults. Scale bar=200 μm. **B** Mean fluorescence intensity (MFI) of FITC in the cortex of each group at 3 d post-HI (n = 18 per group from 3 mice). * $P < 0.05$, *** $P < 0.001$ according to Wilcoxon test. **C** MFI of FITC in the hippocampus of each group at 3 d post-HI (n = 3 per group from 3 mice). *** $P < 0.001$ according to one-way ANOVA with post hoc Bonferroni. **D** Representative VEGFA staining of the brain in each group was performed at 3 days post-HI. Scale bar=100 μm. **E** Quantitative analysis of $VEGFA^+$ cells number in each group (n = 18 per group from 3 mice). *** $P < 0.001$ according to one-way ANOVA with post hoc Dunnett's.

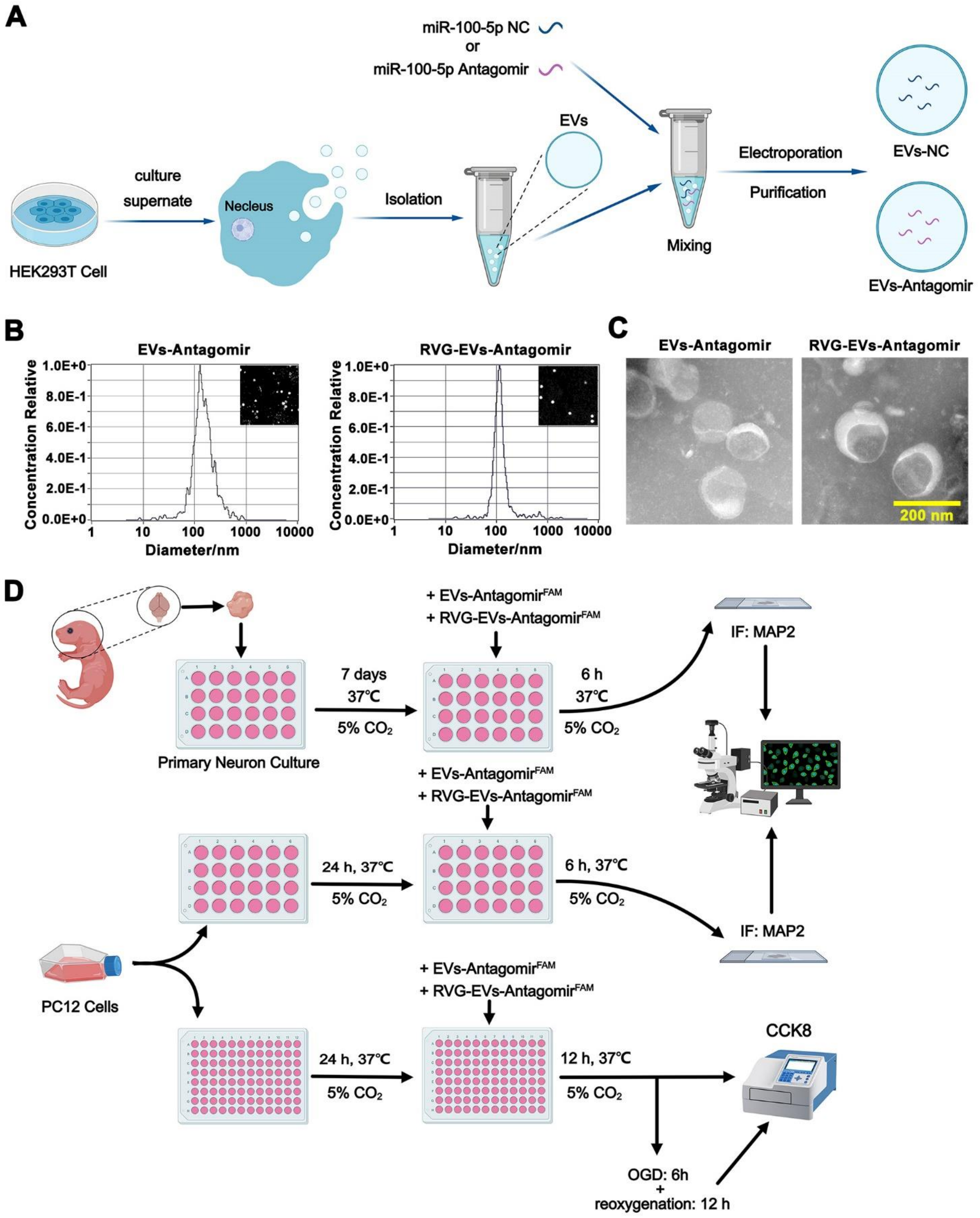

Fig. S5 Flowcharts for production of engineered EVs, its physical characterization, uptake and cytotoxicity test *in vitro*. **A** Schematic (created with MedPeer: www.medpeer.cn) of the EVs loaded with miR-100-5p antagomir extraction process. **B** Size distribution of the isolated EVs-Antagomir and RVG-EVs-Antagomir tested using a nanoparticle tracking analysis. **C** Morphological analysis of EVs-

Antagomir and RVG-EVs-Antagomir by TEM. Scale bar=200 nm **D** Schema (created with MedPeer: www.medpeer.cn) for engineered EVs (EVs-Antagomir^{FAM} and RVG-EVs-Antagomir^{FAM}) uptake assay in primary neuron and PC12 cells; cytotoxicity was assessed using CCK8 in PC12 cells.

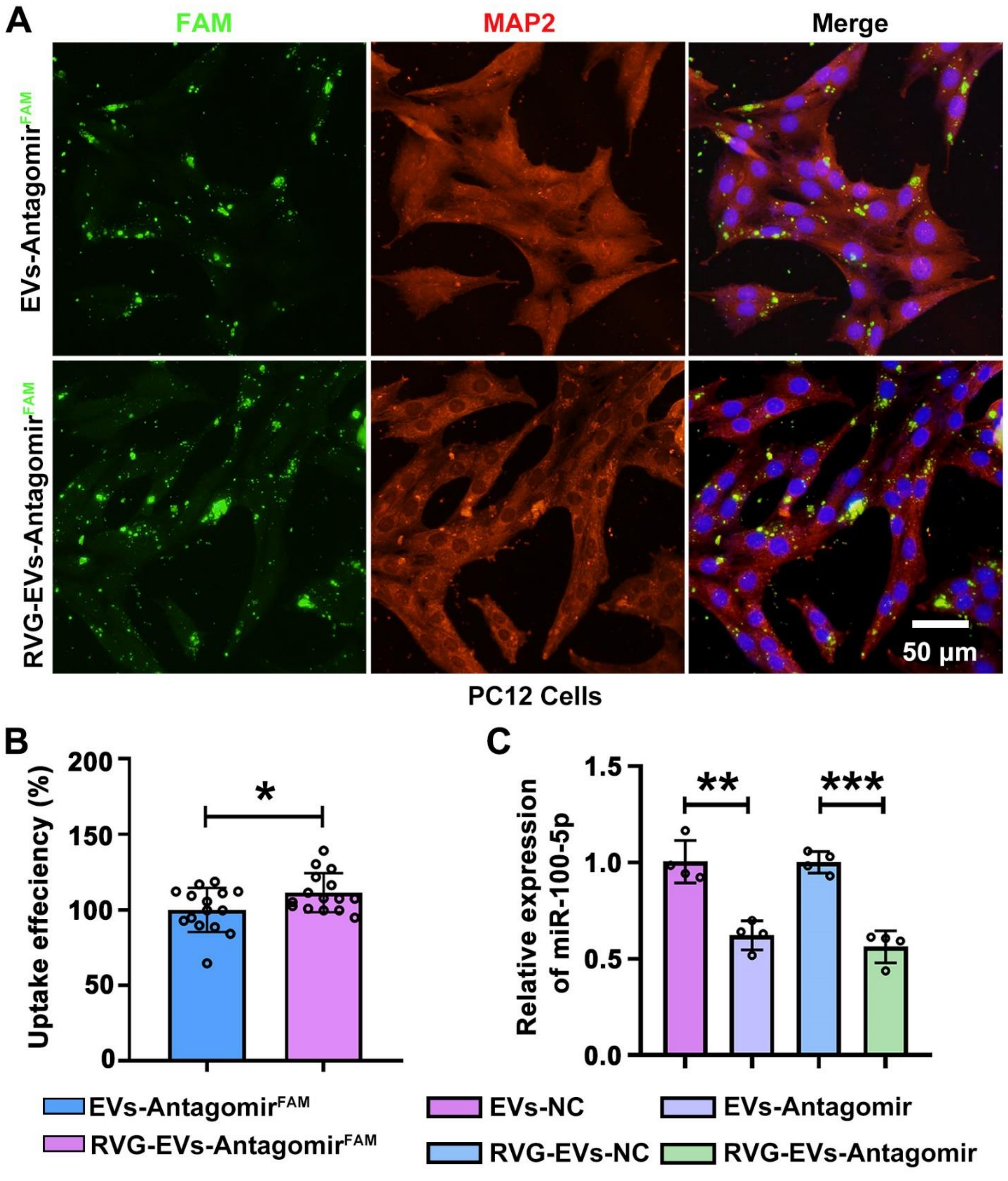

Fig. S6 Engineered EVs could take up by PC12 cells in culture and significantly decreased levels of miR-100-5p. **A** Representative immunofluorescence images of the fluorescent signal of labeled EVs

in PC12 cells. Scale bar=50 μm. **B** Quantification of PC12 cells exosomes signals internalization showed significant increase in the RVG-EVs-Antagomir^{FAM} group compared to EVs-Antagomir^{FAM} group. 5 fields of each cell climbing slice (n = 3) were quantified. **C** The knockdown efficiency of engineered EVs against miR-100-5p in PC12 cells. Experiments were repeated 4 times independently. All data are represented as mean ± SD. * $P < 0.05$, ** $P < 0.01$, *** $P < 0.001$ according to Student′s t-test.

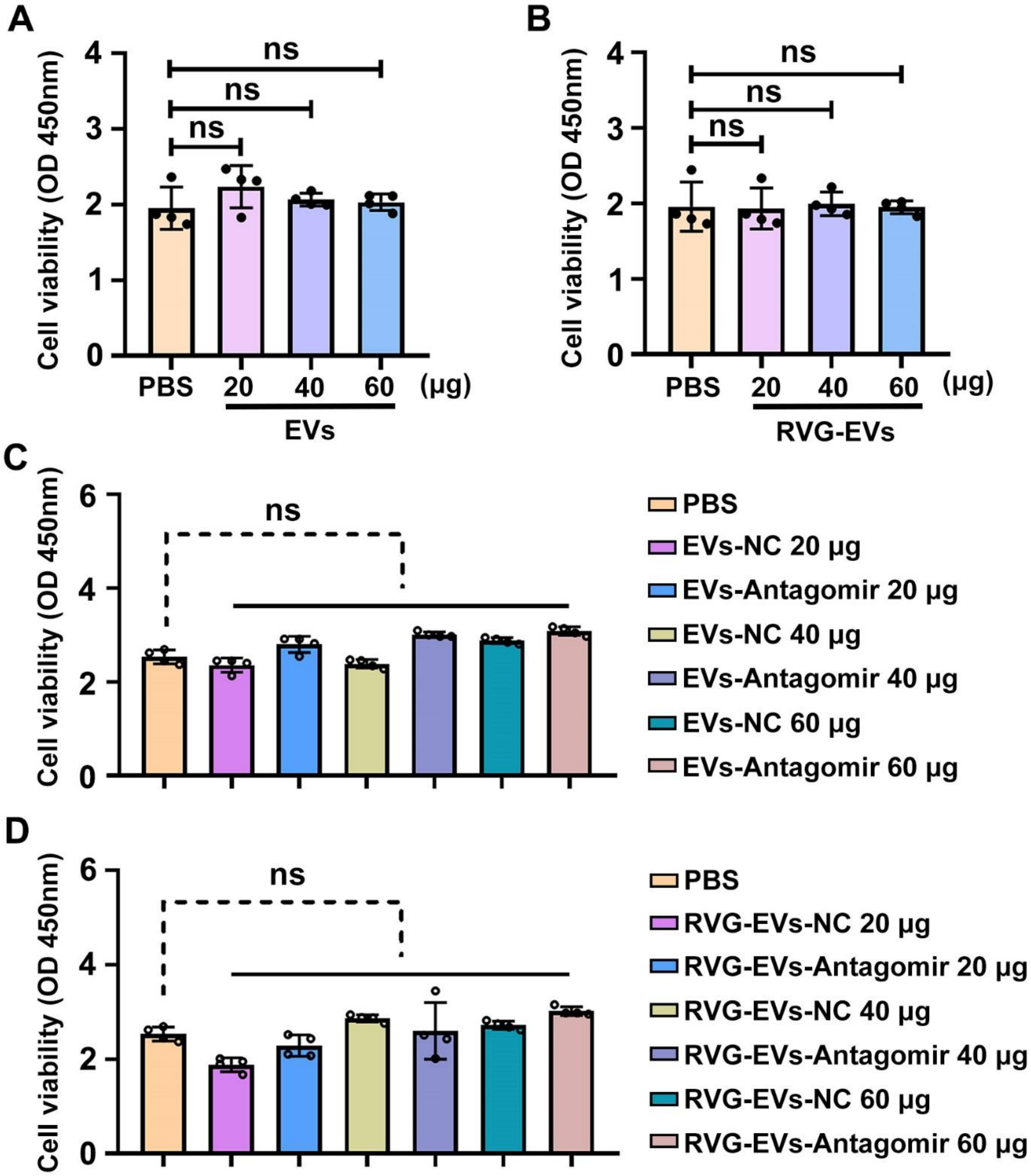

Fig. S7 Cytotoxicity of Engineered EVs. **A** Cytotoxicity of naive EVs derived from HEK293T cells was evaluated by CCK8 assay in PC12 cells. **B** Cytotoxicity of alone RVG-EVs derived from HEK293T cells with Lamp2b-RVG plasmid transfection was assessed by CCK8 in PC12 cells. **C**-**D** Effect of different concentrations of engineered EVs on PC12 cell viability. Experiments were repeated four independent times. All data are represented as mean ± SD, and performed by one-way ANOVA.

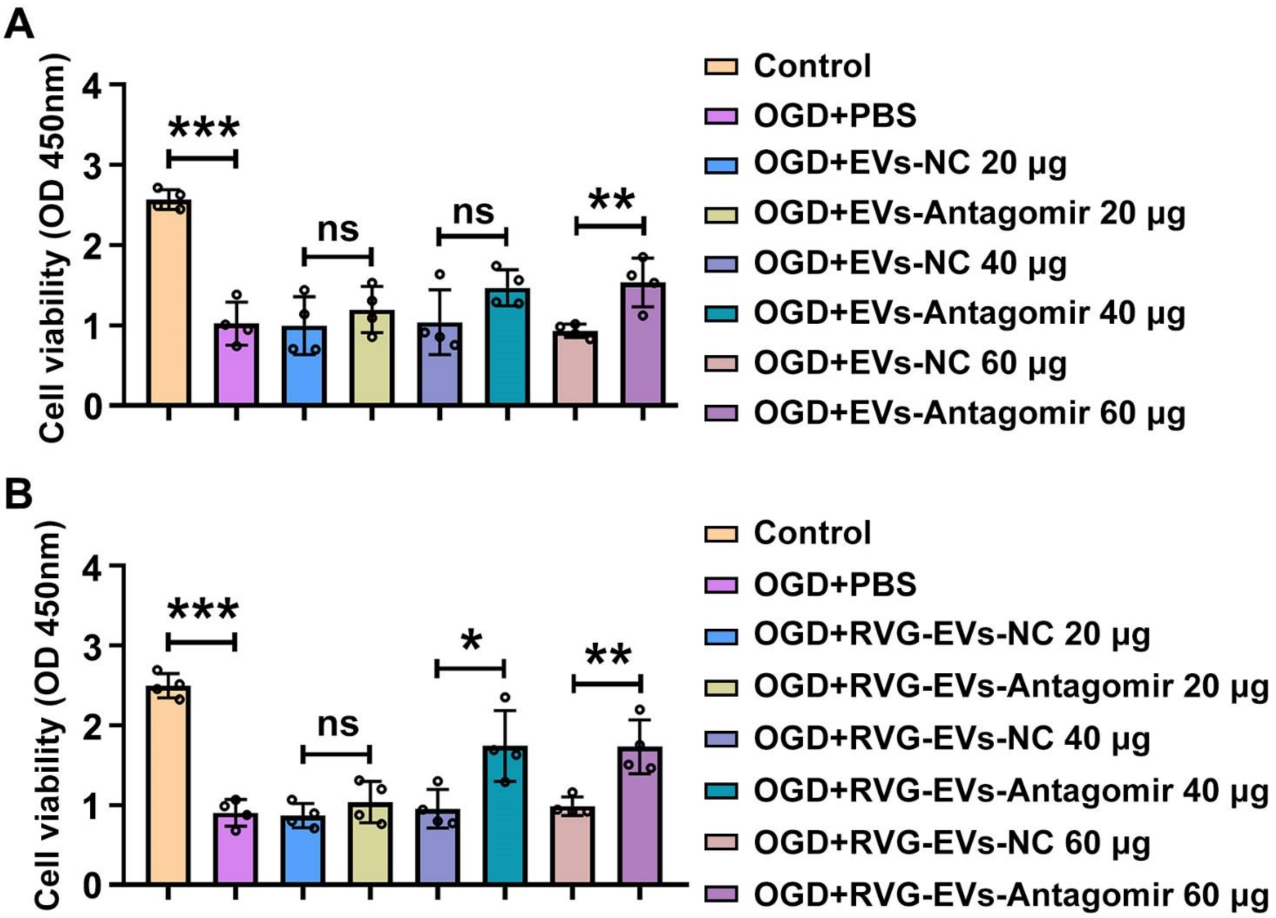

Fig. S8 Effect of different concentrations of engineered EVs on PC12 cell viability after OGD/R exposure. **A**-**B** Cell viability (OD 450 nm) of PC12 cells incubated with different concentrations of engineered EVs was measured by CCK8 after OGD/R exposure (n = 4). All data are represented as mean ± SD. * $P < 0.01$, ** $P < 0.01$, *** $P < 0.001$ according to one-way ANOVA with Bonferroni corrections.

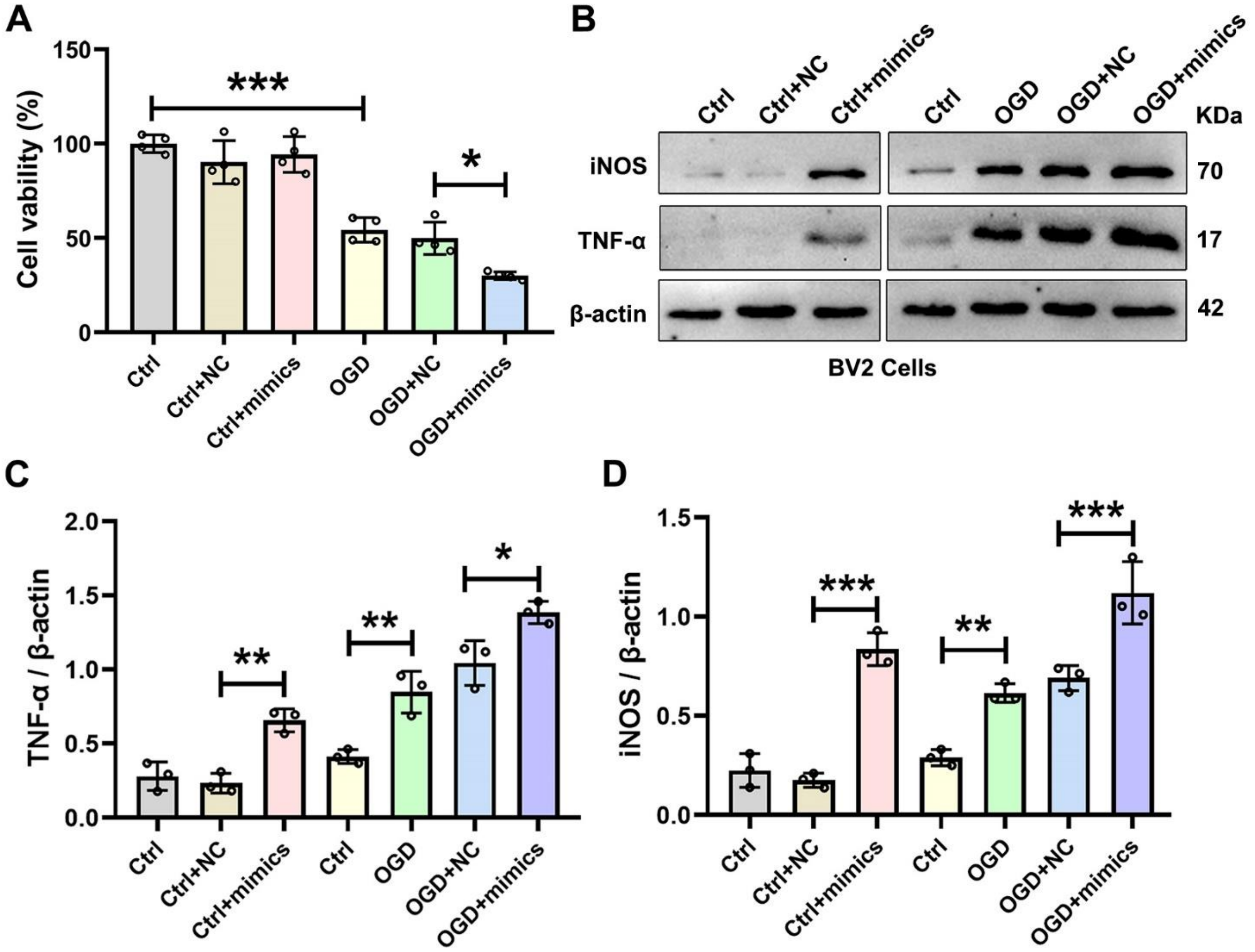

Fig. S9 MiR-100-5p mimics aggravated OGD-induced neuronal apoptosis and neuroinflammation. **A** Cell viability (OD 450 nm) of PC12 cells transfected with miR-100-5p mimics and negative control was measured by CCK8 after OGD/R exposure (n = 4). * $P < 0.01$, *** $P < 0.001$ according to one-way ANOVA with Bonferroni corrections. **B** Western blot was used to analyze relative protein levels of iNOS and TNF-α in BV2 cell lysates of different groups. **C-D** Relative protein levels of TNF-α and iNOS in BV2 cell lysates of different groups (n = 3). Data were normalized to β-actin. * $P < 0.05$, ** $P < 0.01$, *** $P < 0.001$ according to one-way ANOVA followed by Bonferroni post-hoc tests. All data are represented as mean ± SD.

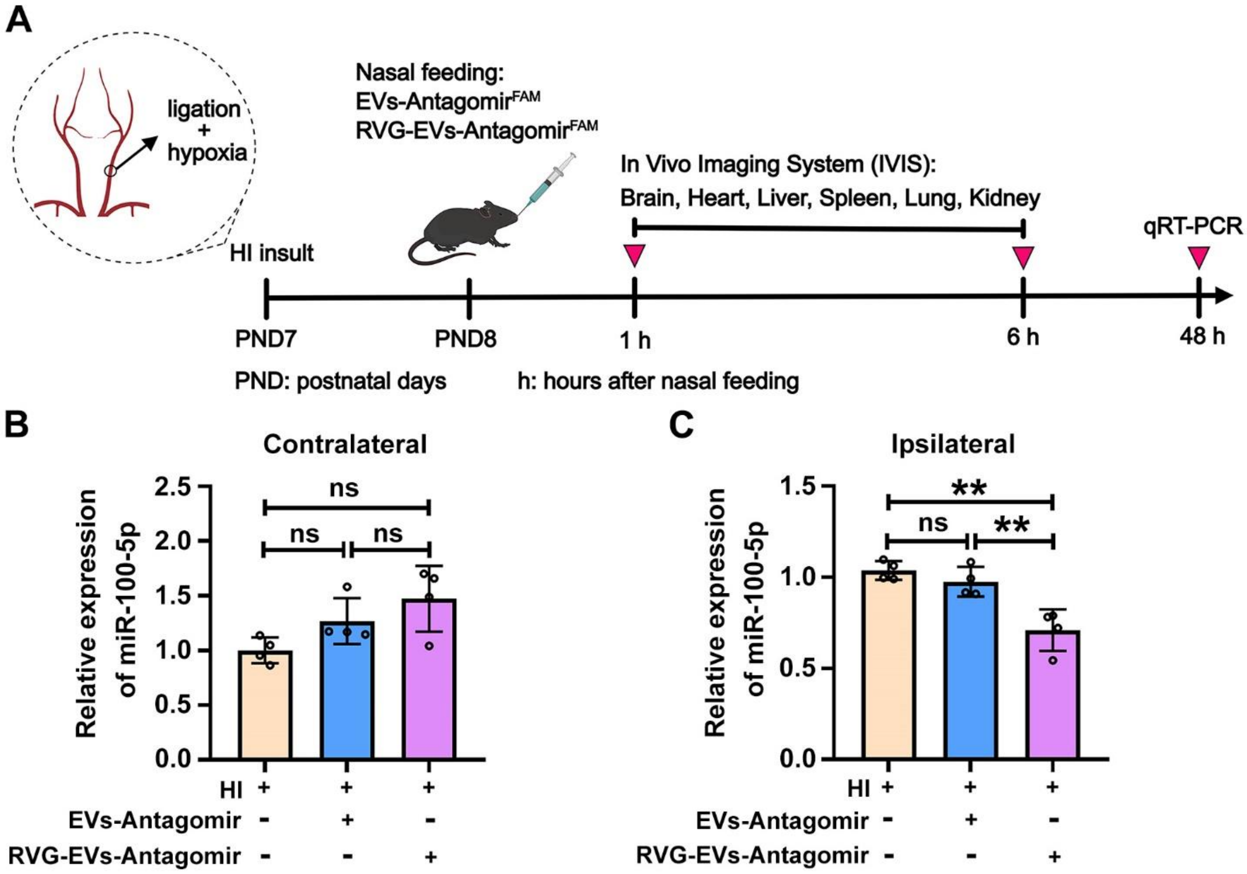

Fig. S10 The knockdown efficiency of engineered EVs against miR-100-5p in the brain tissue of HI mice. **A** Experimental design and timeline for detection of EVs distribution in vivo of HI mice (created with MedPeer: www.medpeer.cn). **B** Expression of miR-100-5p in contralateral cortical tissue of HI mice at 48 h after administration of engineered EVs (n = 4). **C** Expression of miR-100-5p in ipsilateral cortical tissue of HI mice at 48 h after administration of engineered EVs (n = 4). All data are represented as mean ± SD. ** $P < 0.01$ according to one-way ANOVA with Bonferroni corrections.

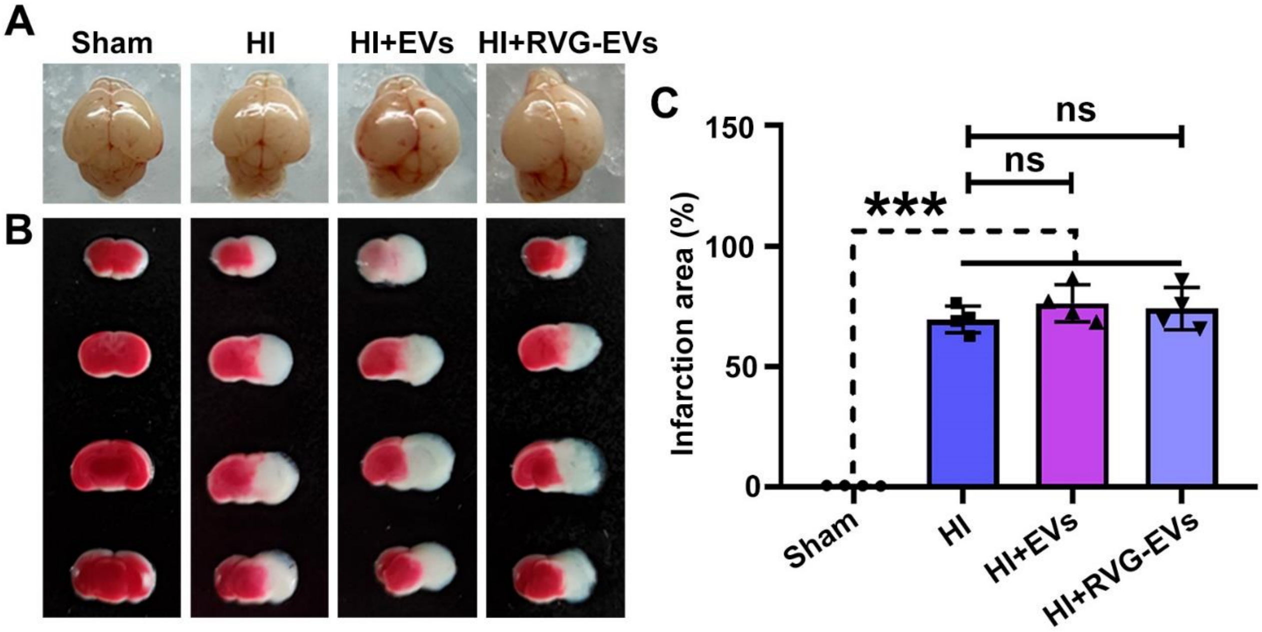

Fig. S11 Administration of EVs and RVG-EVs alone did not improve cerebral edema and infarction caused by HI insult. **A** Representative brain edema images were obtained from each group at 3 d post HI via EVs or RVG-EVs treatment. **B** Representative samples stained with TTC. **C** Quantitative analysis of the infarct area in lesion areas in each group (n = 4). All data are represented as mean ± SD. *** $P < 0.001$ according to One-way ANOVA with Bonferroni post-hoc.

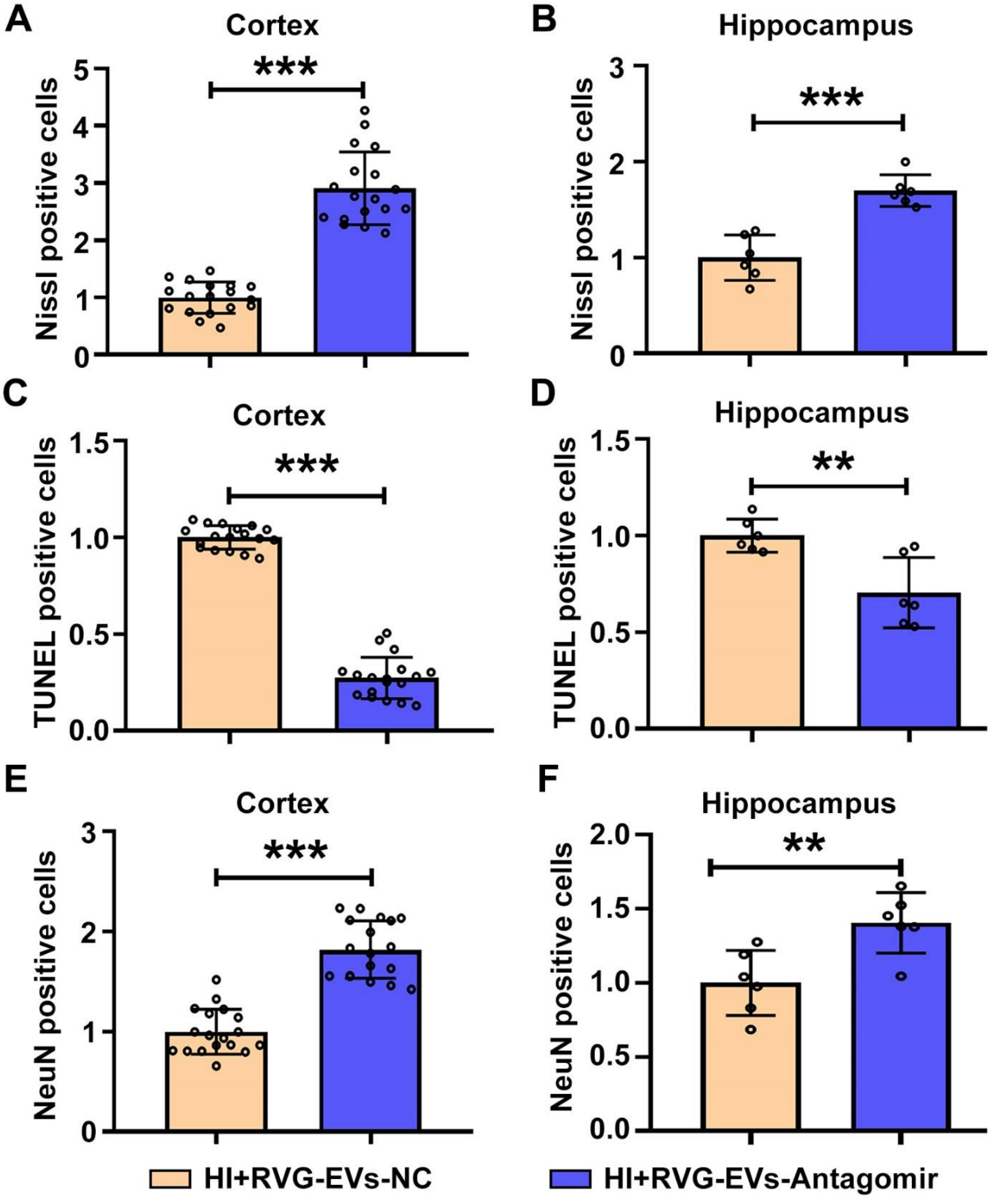

Fig. S12 Administration of RVG-EVs-Antagomir promoted neuronal survival in HI mice. A The ratio of Nissl positive cells in ipsilateral cortex at 3 d after HI with RVG-EVs-Antagomir administration. **B** The ratio of Nissl positive cells in ipsilateral hippocampus at 3 d after HI with RVG-EVs-Antagomir administration. **C** The ratio of TUNEL positive cells in ipsilateral cortex at 3 d after HI with RVG-EVs-Antagomir injection. **D** The ratio of TUNEL positive cells in ipsilateral hippocampus at 3 d after HI with RVG-EVs-Antagomir injection. **E** The ratio of NeuN positive cells

in ipsilateral cortex at 3 d after HI with RVG-EVs-Antagomir treatment. **F** The ratio of NeuN positive cells in ipsilateral hippocampus at 3 d after HI with RVG-EVs-Antagomir treatment. Statistical analysis was derived from three animals per group. All data are represented as mean ± SD. ** $P < 0.01$, *** $P < 0.001$ according to Student′s t-test.

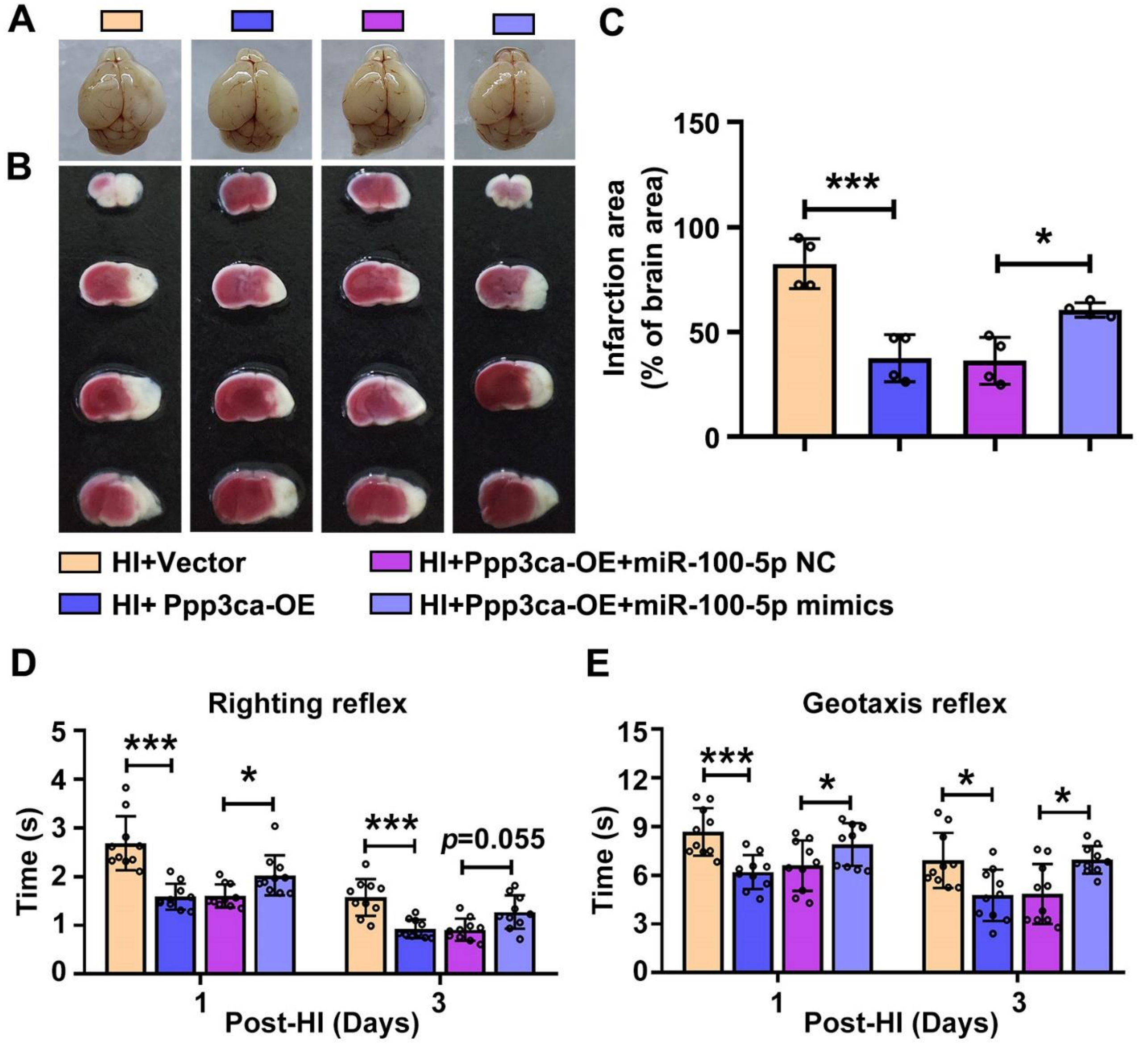

Fig. S13 MiR-100-5p mimics abolished the neuroprotection by Ppp3ca overexpression in HI mice. **A** Representative brain pictures at 3 days following HI in each group treated with Vector, Ppp3ca-OE, Ppp3ca-OE+miR-100-5p NC and Ppp3ca-OE+miR-100-5p mimics. **B** Example of images of TTC staining at 3 d following HI in each group treated with Vector, Ppp3ca-OE, Ppp3ca-OE+miR-100-5p NC and Ppp3ca-OE+miR-100-5p mimics. **C** Quantification of brain infarct area at 3 d following HI

treated with Vector, Ppp3ca-OE, Ppp3ca-OE+miR-100-5p NC and Ppp3ca-OE+miR-100-5p mimics (n = 4 per group), * P < 0.05, *** P < 0.001 according to One-way ANOVA with Bonferroni corrections. **D-E** Short-term neurobehavioral outcome was evaluated via the righting reflex, geotaxis reflex carried out on d 1, d 3 post-HI (n = 10 per group). * P < 0.05, *** P < 0.001 according to Wilcoxon test with Bonferroni corrections at d 1 in **D** and **E**; * P < 0.05, *** P < 0.001 according to One-way ANOVA with Bonferroni corrections at d 3 in **D** and **E**. All data are represented as mean ± SD.

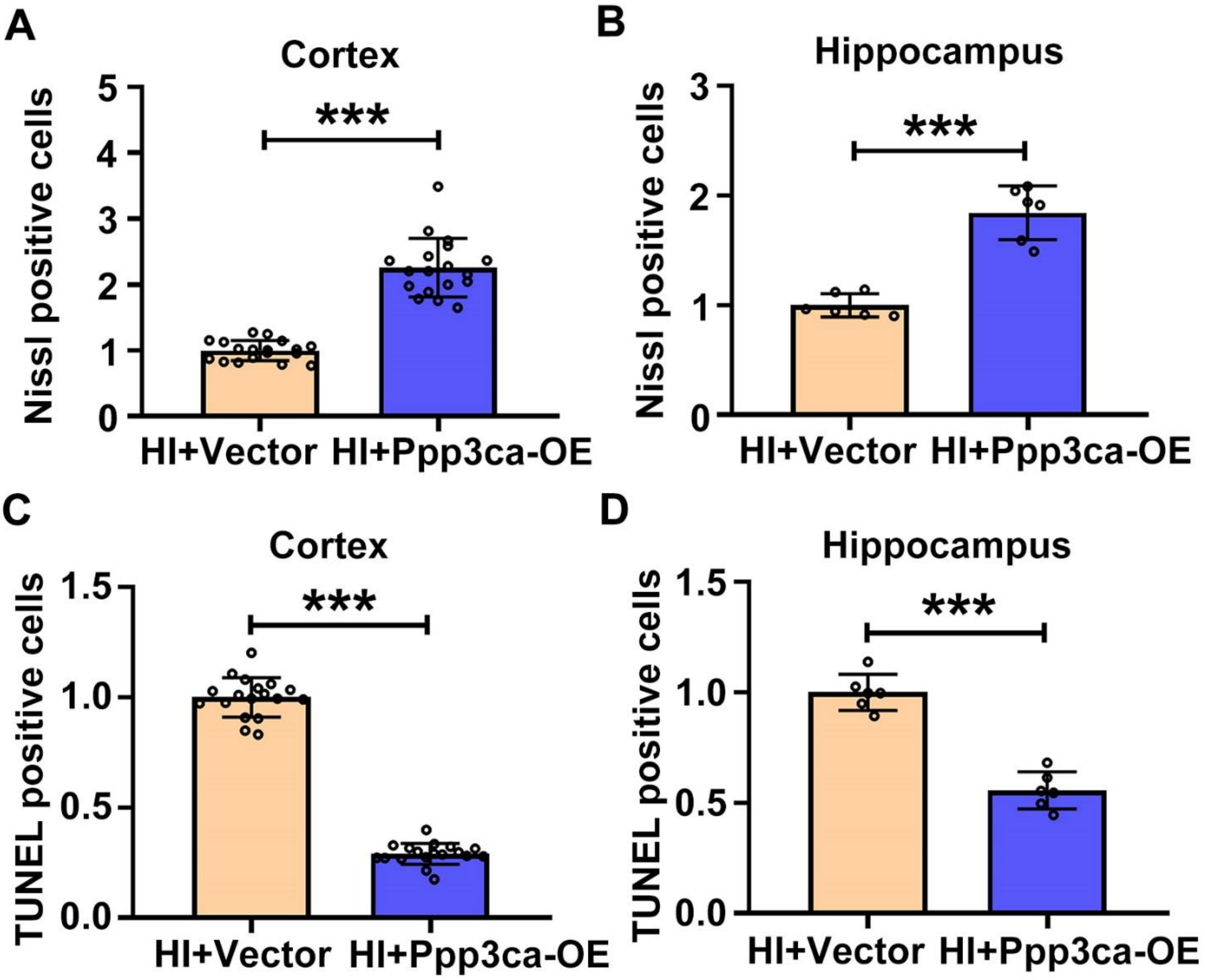

Fig. S14 Overexpression of Ppp3ca alleviates neuronal apoptosis and neuronal loss in HI mice. **A** The ratio of Nissl positive cells in ipsilateral cortex at 3 d after HI with Ppp3ca overexpression plasmid treatment. **B** The ratio of Nissl positive cells in ipsilateral hippocampus at 3 d after HI with Ppp3ca overexpression plasmid treatment. **C** The ratio of TUNEL positive cells in ipsilateral cortex at 3 d after

HI with Ppp3ca overexpression plasmid treatment. **D** The ratio of TUNEL positive cells in ipsilateral hippocampus at 3 d after HI with Ppp3ca overexpression plasmid treatment. Statistical analysis was derived from three animals per group. All data are represented as mean ± SD. *** $P < 0.001$ according to Student′s t-test.

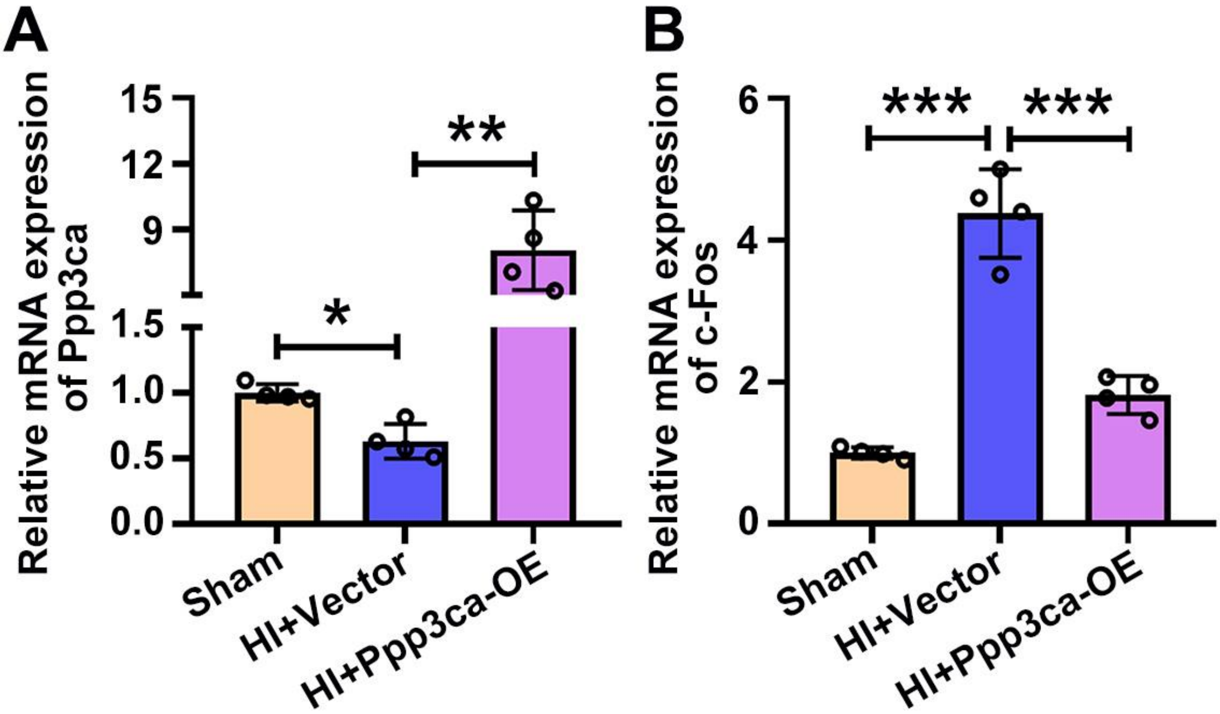

Fig. S15 Overexpression of Ppp3ca reverses the elevation of c-Fos induced by HI. **A** The expression of *Ppp3ca* mRNA was measured in ipsilateral cortex at 3 d after HI with Ppp3ca overexpression plasmid treatment ($n = 4$). * $P < 0.05$, ** $P < 0.01$ according to One-way ANOVA with Bonferroni post-hoc. **B** The expression of *c-Fos* mRNA was measured in ipsilateral cortex at 3 d after HI with Ppp3ca overexpression plasmid treatment ($n = 4$). *** $P < 0.001$ according to One-way ANOVA with Dunnett's post-hoc. All data are represented as mean ± SD.

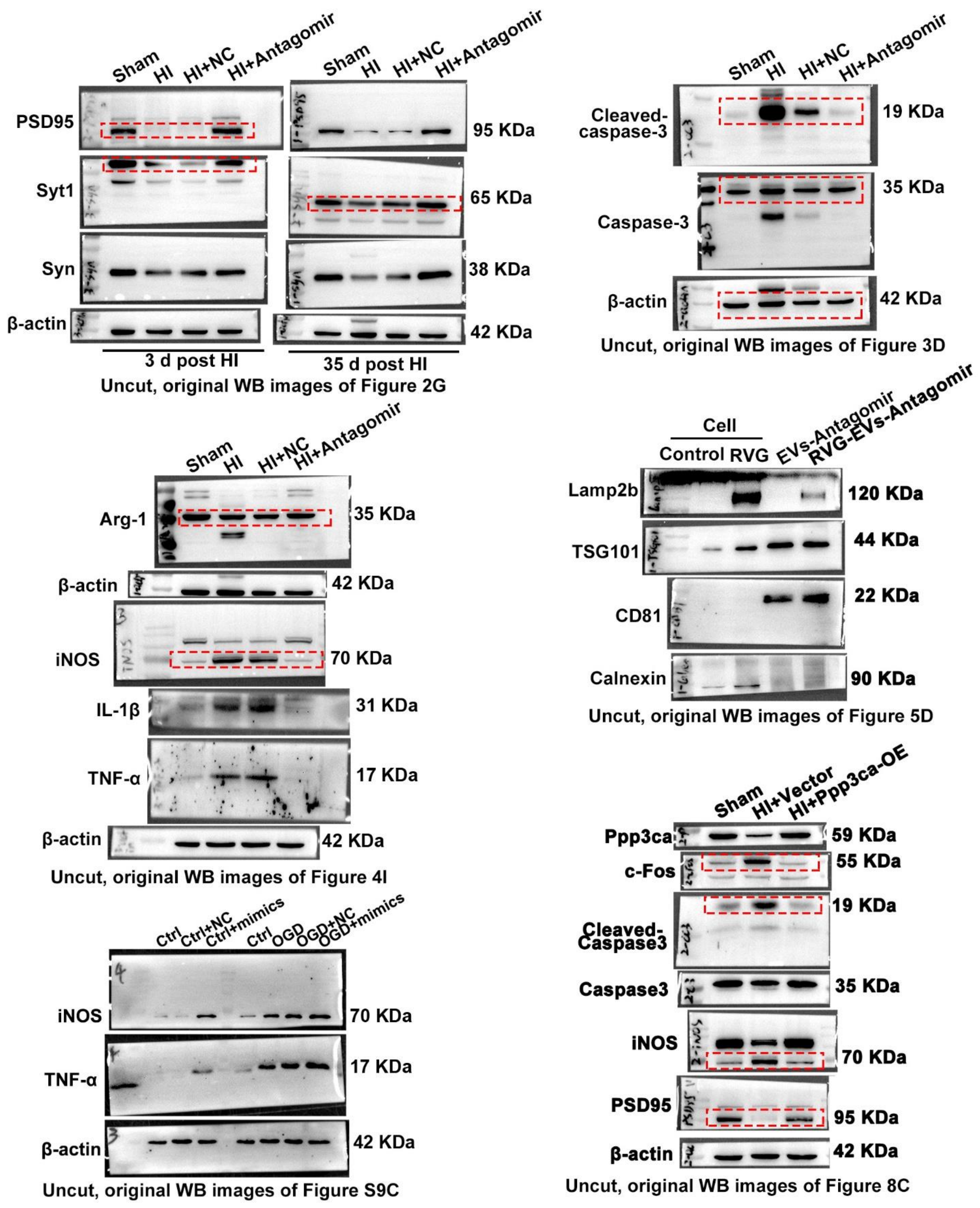

Fig. S16 The uncropped images of western blot data.
